# Dearomatized Isoprenylated Acylphloroglucinol Derivatives with Potential Antitumor Activities from *Hypericum henryi*

**DOI:** 10.1007/s13659-019-00229-w

**Published:** 2020-02-04

**Authors:** Yan-Song Ye, Man Wu, Na-Na Jiang, Yuan-Zhi Lao, Wen-Wei Fu, Xia Liu, Xing-Wei Yang, Juan Zhang, Hong-Xi Xu, Gang Xu

**Affiliations:** 1 State Key Laboratory of Phytochemistry and Plant Resources in West China and Yunnan Key Laboratory of Natural Medicinal Chemistry, Kunming Institute of Botany, Chinese Academy of Sciences, Kunming, 650201 China; 2 School of Pharmacy, Shanghai University of Traditional Chinese Medicine, Shanghai, 201203 China; 3 Department of Pharmacy, Chongqing Traditional Chinese Medicine Hospital, Chongqing, 400021 China; 4 University of Chinese Academy of Sciences, Beijing, 100049 China

**Keywords:** *Hypericum henryi*, Dearomatized isoprenylated acylphloroglucinols (DIAPs), Apoptosis, Cell cycle arrest

## Abstract

A series of dearomatized isoprenylated acylphloroglucinols derivatives, hyperhenols A–E (**1**–**5**), as well as seven known analogues (**6**–**12**), were characterized from *Hypericum henryi*. Their structures were determined by combination of NMR, ECD spectroscopy, and X-ray diffraction analysis. Compounds **1** and **6**–**8** were tested to exhibit potential antitumor properties, of which **6** and **7** inhibited cell growth through inducing apoptosis and cell cycle arrest. In addition, these compounds could induce autophagy and PINK1/Parkin-mediated mitophagy in cancer cell lines, as well as suppress lung cancer A549 cells metastasis in vitro.

**Electronic supplementary material:**

The online version of this article (10.1007/s13659-019-00229-w) contains supplementary material, which is available to authorized users.

## Introduction

Natural phloroglucinol derivatives are widely distributed in Myrtaceae, Guttiferae, Euphorbiaceae, Aspidiaceae families as well as appeared in marine and microbial sources [1]. In which prenylated acylphloroglucinols are a special kind of hybrid natural products originated from a polyketide combined isoprenylation biosynthetic pathways, and were mainly reported from the plants of genus *Hypericum* and *Garcinia* in the family Guttiferae [2–4]. With their wide range of biological profiles and diverse molecular architectures exemplified by hyperforin [5], hypersubone B [6], hyperuralone A [7] and chinesins Ι/ΙΙ [8], prenylated acylphloroglucinol derivatives have attracted great interest of chemists and pharmaceutists.

**Fig. 1:**
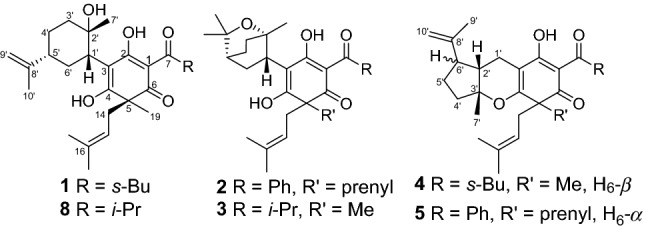
Structures of compounds **1**–**5**

As a traditional folk medicine in China, *Hypericum henryi* has been used to treat hepatitis [9]. Previous investigations on this plant have reported structurally diverse polycyclic polyprenylated acylphloroglucinols (PPAPs) such as hyphenrones A–X [10–12]. As a part of systematic search on bioactive acylphloroglucinol derivatives, five new dearomatized isoprenylated acylphloroglucinols (DIAPs) derivatives, hyperhenols A–E (**1**–**5**) together with seven known analogues (**6**–**12**) were isolated from *H. henryi* (Fig. 1). In the bioactive study, compounds **1** and **6**–**8** were found to exhibit promising cytotoxic activities against three human cancer cell lines in vitro. And the further studies indicated compounds **6** and **7** could trigger autophagy, PINK1/Parkin-mediated mitophagy in cancer cell lines, and also suppress lung cancer A549 cells metastasis targeting Akt and cofilin signaling pathways. In addition, **6** and **7** also displayed significant anti-proliferation activities by inducing apoptosis and cell cycle arrest. Herein, the isolation, structure elucidation, and bioactivities evaluation of these compounds were reported.

## Results and Discussion

The MeOH extract was subjected to repeated column chromatography to yield five new DIAPs derivatives (**1**–**5**) together with seven known analogues hyphenrone J (**6**) [13], hyphenrone K (**7**) [13], hyperhenone E (**8**) [12], hyperhenone A (**9**) [12], hyperhenone B (**10**) [12], hyperhenone C (**11**) [12], and hyperhenone D (**12**) [12].

**Fig. 2:**
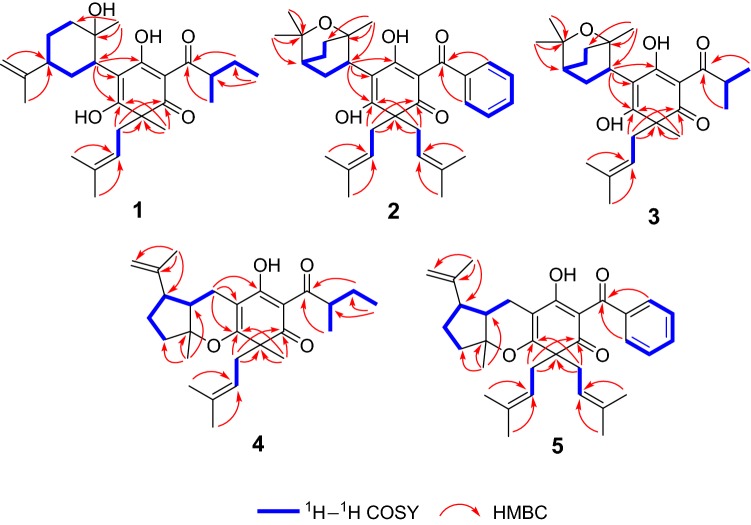
Key HMBC and ^1^H–^1^H COSY correlations of **1**–**5**

Hyperhenol A (**1**) was isolated as yellow oil and assigned molecular formula of C_27_H_40_O_5_ with 8 degrees of unsaturation by HRESIMS (*m/z* 443.2803 [M − H]^−^, calcd. C_27_H_39_O_5_, 443.2803). The IR spectrum displayed bands for hydroxy (3417 cm^−1^) and carbonyl groups (1636 cm^−1^). The ^13^C NMR data along with DEPT experiments showed 27 carbon signals including seven methyls, six methylenes, four methines, and ten quaternary carbons (three oxygenated tertiary carbons and two carbonyls). Detailed analysis of the ^13^C NMR spectroscopic data (Table 1) indicated the presence of an isoprenyl (*δ*_C_ 40.3, C-14; 120.5, C-15; 135.3, C-16; 26.2, C-17; 18.2, C-18), a DIAPs core including an enolized 1,3-diketone group (*δ*_C_ 199.0, C-6; 107.4, C-1; 191.0, C-2), an enolic double bonds (*δ*_C_ 111.1, C-3; 179.4, C-4), a carbonyl (*δ*_C_ 207.9, C-7), and a quaternary carbon at *δ*_C_ 54.7 (C-5) [12, 13]. The location of the mentioned isoprenyl and the methyl (*δ*_C_ 24.6, C-19) at C-5 was evidenced by the HMBC correlations from H_2_-14 (*δ*_H_ 2.62, 2.56) and Me-19 (*δ*_H_ 1.32) to C-4 (*δ*_C_ 179.4), C-5 (*δ*_C_ 54.7) and C-6 (*δ*_C_ 199.0) (Fig. 2). Besides the aforementioned DIAP moiety, the remaining 14 carbon signals can be attributed to a *sec*-butyl group (*δ*_C_ 43.4, C-8; *δ*_C_ 17.1, C-9; *δ*_C_ 27.7, C-10; *δ*_C_ 12.4, C-11) and another C_10_ unit.

**Table 1:** The ^13^C (150 MHz) NMR data of compounds **1**–**5** (*δ* in ppm)

No	1^a^	2^b^	3^a^	4^b^	5^b^
1	107.4	107.5	106.3	106.5	108.2
2	191.0	189.0	190.8	189.2	188.8
3	111.1	112.4	112.1	103.3	103.3
4	179.4	176.6	177.4	170.6	171.2
5	54.7	57.6	54.6	52.4	57.0
6	199.0	195.3	198.4	196.4	195.2
7	207.9	195.5	208.5	206.8	196.3
8	43.4	139.2	36.8	41.7	139.3
9	17.1	127.6	19.2	16.4	127.8
10	27.7	127.7	19.5	26.8	128.0
11	12.4	130.6		11.8	131.0
12		127.7			128.0
13		127.6			127.8
14	40.3	38.5	38.8	38.0	37.3
15	120.5	119.0	120.7	118.8	119.3
16	135.3	133.7	135.2	134.2	134.0
17	26.2	18.1	18.2	25.9	26.2
18	18.2	25.9	26.1	18.1	26.2
19	24.6	38.0	25.6	24.0	38.4
20		119.3			118.8
21		134.0			134.4
22		25.9			18.5
23		18.1			18.2
1′	42.7	35.1	36.2	35.5	16.3
2′	73.3	74.8	76.0	40.5	43.3
3′	41.0	33.2	34.2	86.8	88.4
4′	27.9	21.2	22.2	15.2	27.1
5′	47.1	32.8	33.9	22.2	37.8
6′	32.2	31.0	32.3	47.0	49.1
7′	28.0	23.5	24.2	26.9	23.0
8′	151.0	78.0	79.3	143.5	145.7
9′	109.2	28.3	27.2	23.8	19.4
10′	21.1	26.6	28.6	111.6	111.8

^a^Recorded in CD_3_OD

^b^Recorded in CDCl_3_

Comparison of ^1^H and ^13^C NMR data indicated that the C_10_ unit of **1** shared a similar structure with the monoterpenoid moiety of callistrilone B [14], which was confirmed by the ^1^H–^1^H COSY correlations of H-3′/H-4′/H-5′/H-6′/H-1′, accompanied by HMBC correlations from Me-7′ (*δ*_H_ 1.18) to C-1′ (*δ*_C_ 42.7), C-2′ (*δ*_C_ 73.3), and C-3′ (*δ*_C_ 41.0); from Me-10′ (*δ*_H_ 1.58) to C-8′ (*δ*_C_ 151.0), C-5′ (*δ*_C_ 47.1), and C-9′ (*δ*_C_ 109.2); and from H_2_-9′ (*δ*_H_ 4.71, 4.67) to C-8′ (*δ*_C_ 151.0), C-10′ (*δ*_C_ 21.1), and C-5′ (*δ*_C_ 47.1). The linkage of DIAP core and monoterpenoid moiety between C-1′ and C-3 was evidenced by HMBC correlations from H-1′ (*δ*_H_ 3.24) to C-2 (*δ*_C_ 191.0), C-3 (*δ*_C_ 111.1), and C-4 (*δ*_C_ 179.4) (Fig. 2). Finally, the *sec*-butyl group only can be attached at C-1.

**Fig. 3:**
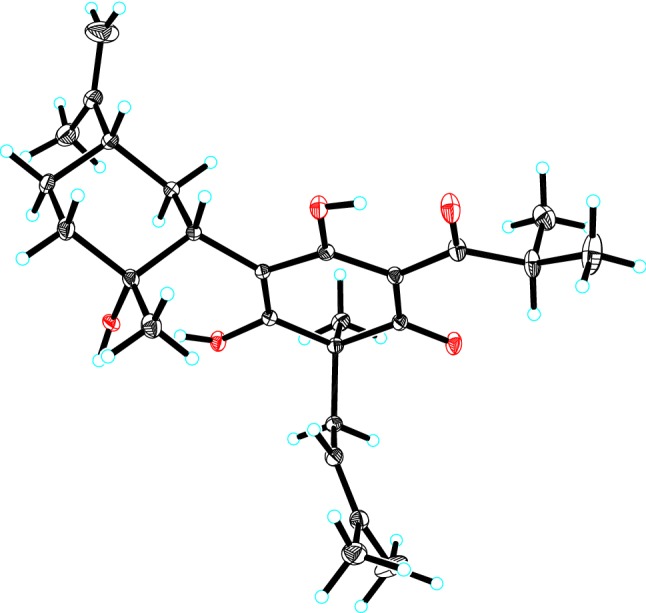
X-ray structure of compound **8**

**Fig. 4:**
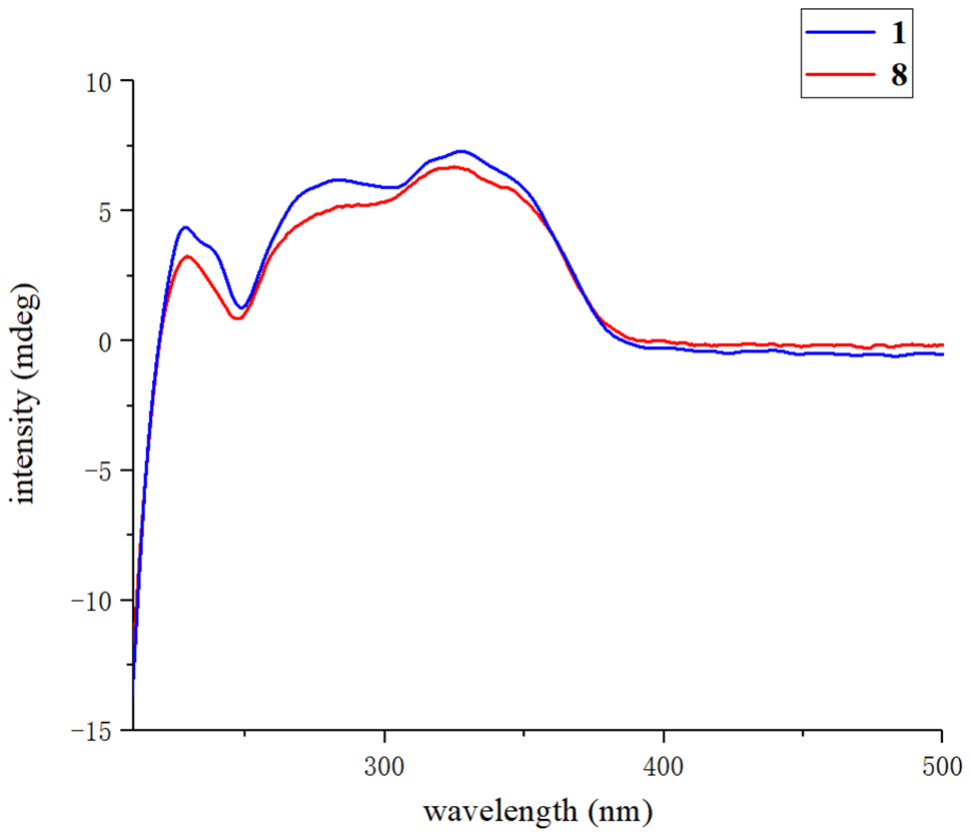
Experimental ECD spectra of **1** and **8**

The relative configuration of monoterpenoid moiety was determined by detailed interpretation of the ROESY spectrum. The NOE correlations of H-5′/H-1′ and H-1′/Me-7′ indicated that H-5′, H-1′ and Me-7′ were on the same side. However, due to the rotation of carbon–carbon single bond (C-3/C-1′) between DIAP core and monoterpenoid, determination of the configuration of C-5 is still challenging. For instance, an analogue of **1** (hyperhenone E, **8**), has also been reported with the configuration of C-5 undefined [12]. In this study, hyperhenone E, as well as its crystals, was fortunately obtained, which unambiguously determined absolute configurations of **8** as 5*S*, 1′*R*, 2′*R*, 5′*S* (Fig. 3). Furthermore, the absolute configurations of C-5, C-1′, C-2′ and C-5′ in **1** were also determined to be the same with those of **8** via their well-matched ECD curves (Fig. 4).

**Fig. 5:**
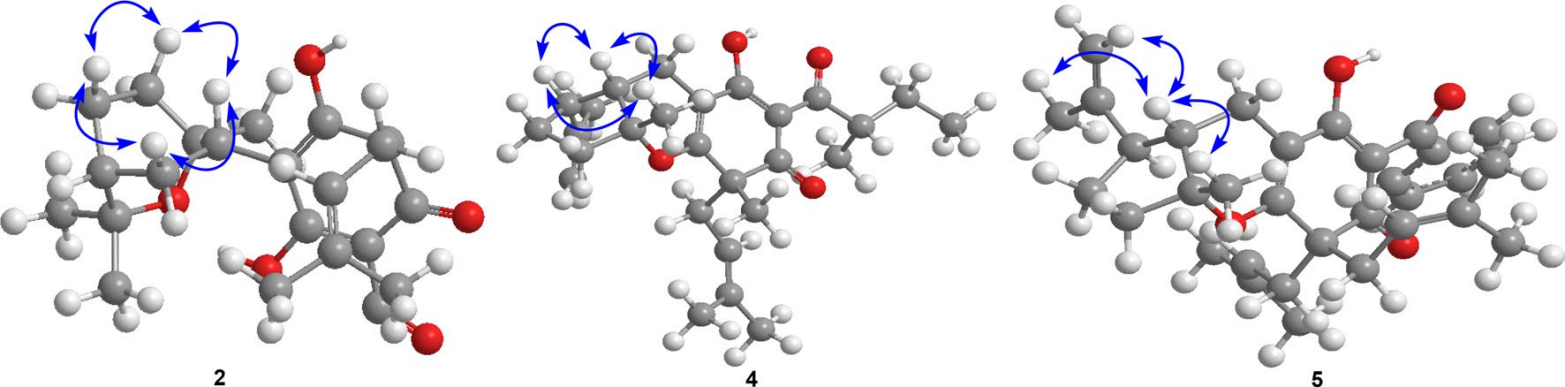
Key ROESY correlations of **2**, **4** and **5**

Hyperhenol B (**2**) was obtained as yellow oil. A molecular formula of C_33_H_42_O_5_, was deduced by its ^13^C NMR and HRESIMS (*m/z* 519.3106 [M + H]^+^, calcd. C_33_H_43_O_5_ 519.3105). The ^1^H and ^13^C NMR spectra of **2** and hyperhenone F are closely similar to each other [12]. Comparative analyses of their NMR data revealed that the isopropyl in hyperhenone F was replaced by a phenyl, which was supported by the HMBC correlations from H-9/H-13 (*δ*_H_ 7.43) to C-7 (*δ*_C_ 195.5) and C-8 (*δ*_C_ 139.2) in **2** (Fig. 2). Because of the rigidity of the bicyclic skeleton, cyclohexane moiety tended to form boat conformation. Hence, the ROESY correlations of H-1′/H-3′ (*δ*_H_ 1.96), H-4′/H-6′ (*δ*_H_ 2.10), and H-6′/ H-1′ showed the same orientations of H-1′ and Me-7′ (Fig. 5).

Hyperhenol C (**3**) exhibited a molecular formula of C_26_H_38_O_5_, as assigned by HRESIMS (*m/z* 429.2653 [M − H]^−^, calcd. C_26_H_37_O_5_, 429.2646). The NMR spectra of **3** showed a close resemblance to those of hyperhenone F except that the signals for the isoprenyl at C-5 in hyperhenone F was replaced by a methyl in **3** [12], which can be further confirmed by the HMBC correlations from Me-19 (*δ*_H_ 1.35) to C-5 (*δ*_C_ 54.6), C-4 (*δ*_C_ 177.4), C-6 (*δ*_C_ 198.4) and C-14 (*δ*_C_ 38.8). The similar NOE correlations of H-1′/H-3′ (*δ*_H_ 1.70), H-3′/H-4′ (*δ*_H_ 2.09), H-4′/H-6′ (*δ*_H_ 1.61) and H-6′/H-1′ showed that relative configurations were the same as those of **2** (Fig. 5). In the absence of sufficient evidence, configuration of C-5 could not be determined.

Hyperhenol D (**4**) was obtained as yellow oil. The molecular formula was established as C_27_H_38_O_4_ based on its HRESIMS data (*m/z* 427.2855 [M + H]^+^, calcd. C_27_H_39_O_4_ 427.2843), implying 9 indices of hydrogen deficiency. The characteristic information for a DIAPs core was clearly observed in the ^13^C NMR spectra (*δ*_C_ 106.5, C-1; *δ*_C_ 189.2, C-2; *δ*_C_ 103.3, C-3; *δ*_C_ 170.6 C-4; *δ*_C_ 52.4 C-5; *δ*_C_ 196.4 C-6). A comparison of the 1D NMR data of **4** with those of chinesin Ι suggested that they shared closely similar plane structures [8]. The molecular formulas (C_27_H_40_O_4_ for chinesin Ι; C_27_H_38_O_4_ for **4**) revealed that **4** possessed one more degree of unsaturation [8], which could derived by the loss of H_2_O between hydroxyls of monoterpenoid and DIAPs core of chinesin Ι to afford **4**. The ether linkage of C-4 and C-3′ was evidenced by indices of hydrogen deficiency, the downfield chemical shift of C-3′ (*δ*_C_ 86.8) and the ROESY correlation of Me-7′/Me-17. The relative configurations of C-2′, C-3′, and C-6′ were elucidated by key ROESY correlations of Me-7′/H-2′, Me-7′/H-6′, and H-2′/H-6′. Unfortunately, the configuration of C-5 also could not be determined since the absence of sufficient evidence.

Hyperhenol E (**5**) was obtained as yellow oil, and its HRESIMS spectrum (*m/z* 501.3008 [M + H]^+^, calcd. C_33_H_41_O_4_ 501.2999) showed a molecular formula of C_33_H_40_O_4_. The ^1^H NMR data of **5** (Table 2) exhibited a monosubstituted benzene (*δ*_H_ H 7.30, 3H; 7.38, 2H), two isoprenyl (*δ*_H_ 4.75, t, *J* = 7.2 Hz; *δ*_H_ 4.81, t, *J* = 7.2 Hz). The NMR spectra of **5** showed a close resemblance to those of **4** except for the replacements of the *sec*-butyl group at C-7 and the methyl at C-5 in **4** by a phenyl and an isoprenyl in **5**, respectively. This conclusion was verified via the ^1^H–^1^H COSY cross peak of H_2_-19/H-20 combined with the HMBC correlations of H_2_-19 (*δ*_H_ 2.57 and 2.38) with C-4 (*δ*_C_ 171.2)/C-5 (*δ*_C_ 57.0)/C-6 and H-9 (*δ*_H_ 7.30)/H-13 (*δ*_H_ 7.30) with C-7 (*δ*_C_ 195.2)/C-8 (*δ*_C_ 108.2). In the ROESY spectrum, the obvious NOE correlation between Me-7′ and H-2′ can also be found as that in **4**, but the diagnostic signals of H-2′/H-6′ and Me-7′/H-6′ in **4** were replaced by the H-2′/Me-9′and H-2′/H-10 (*δ*_H_ 4.62), which indicated that the orientation of H-6′ was different with that of Me-7′/H-2′.

**Table 2:** The ^1^H (600 MHz) NMR data of compounds **1**–**5** (*δ* in ppm and *J* in Hz)

No	**1**^a^	**2**^b^	**3**^a^	**4**^b^	**5**^b^
8	3.87, m		3.98, sept (6.9)	3.86, m	
9	1.09, d (6.6)	7.43, overlap	1.11, d (6.9)	1.10, d (6.9)	7.30, overlap
10	1.81, overlap	7.35, overlap	1.10, d (6.9)	1.68, overlap	7.38, overlap
	1.62, overlap			1.34, m	
11	0.89, m	7.43, overlap		0.89, m	7.38, overlap
12		7.35, overlap			7.38, overlap
13		7.43, overlap			7.30, overlap
14	2.62, m	2.61, overlap	2.63, d (7.8)	2.62, m	2.57, m
	2.56, m			2.41, m	2.47, m
15	4.83, overlap	4.88, overlap	4.78, t (7.2)	4.72, t (7.2)	4.75, t (7.2)
17	1.54, s	1.58, s	1.58, s	1.54, s	1.58, s
18	1.58, s	1.47 s	1.51, s	1.54, s	1.55, s
19	1.32, s	2.61, overlap	1.35, s	1.28, s	2.57, m
					2.38, m
20		4.88, overlap			4.81, t, (7.2)
22		1.37 s			1.53, s
23		2.61, overlap			1.53, s
1′	3.24, m	3.35, m	3.39, m	1.81, m	2.47, d (8.2)
2′				1.93, overlap	2.24, d (8.2)
3′	1.81, overlap	1.96, m	2.00, m		1.92, dd (12.0, 6.5)
	1.62, overlap	1.68, overlap	1.70, m		
4′	1.81, overlap	1.96, m	2.09, m	2.31, m	1.77, overlap
	1.37, m	1.68, overlap	1.73, m	1.68, overlap	
5′	2.09, m	1.47, m	1.61, overlap	1.93, overlap	2.03, ms
				1.68, overlap	1.77, overlap
6′	1.92, m	2.10, m	2.12, m	2.79, m	2.24, m
	1.30, overlap	1.59, m	1.61, overlap		
7′	1.18, s	1.10, s	0.98, s	1.45, s	1.26, s
8′					
9′	4.71, s	1.37, s	1.40, s	1.76, s	1.62, s
	4.67, s				
10′	1.58, s	1.30, s	1.33, s	4.93, s	4.72, s
				4.72, s	4.62, s

^a^Recorded in CD_3_OD

^b^Recorded in CDCl_3_

**Fig. 6:**
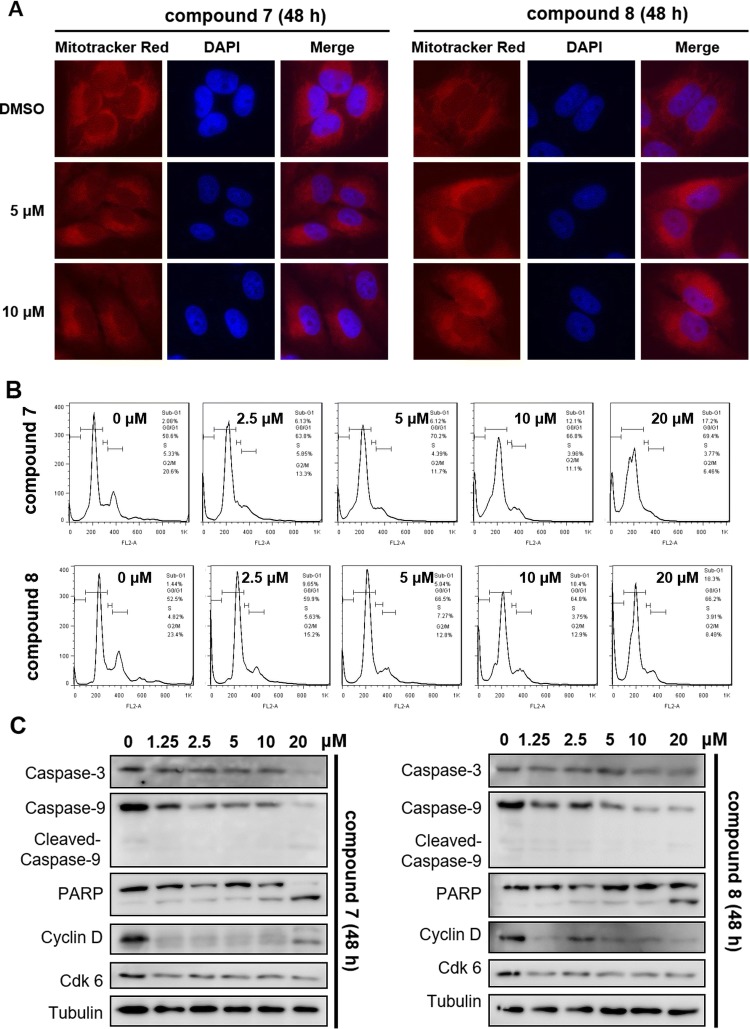
Effects of compounds on apoptosis and cell cycle. **a** HeLa cells were stained with MitoTracker red (100 nM) and analyzed by fluorescence microscope. Scale bars: 10 *μ*M. **b** Cell cycle analysis in Hela cells after 48 h. **c** Apoptotic and cell cycle related proteins were analyzed by western blot

In the searching for their anticancer properties, compounds **1** and **6**–**8** were found to effectively inhibit cell growth in HeLa, A549, and MDAMB-231 cell lines (Table 3). Of which **6** and **7** could significant inhibit cancer cells growth with the IC_50_ up to 0.07 and 0.09 *μ*M, respectively. Both the two compounds could also obviously increase mitochondrial fission and further activated the caspase-3, caspase-9, and increased PARP cleavage in HeLa cells (Fig. 6a, c). Treatment with **6** and **7** also increased the percentage of cells in G0/G1 phase and decreased in G2/M phase (Fig. 6b). Moreover, western blot results indicated that these two compounds efficiently suppressed the expression of cyclin D1 and Cdk 6 in HeLa cells, suggesting **6** and **7** induced cell cycle arrest. (Fig. 6c). Taken together, these results demonstrated that these compounds inhibited cell growth through inducing apoptosis and cell cycle arrest.

**Table 3:** Cytotoxicity of the isolates on three cancer cell lines with IC_50_ values (*μ*M)

Compound^a^	HeLa	A549	MDA-MB-231
1	0.88 ± 0.042	3.53 ± 0.074	4.18 ± 0.43
6	0.07 ± 0.04	1.85 ± 0.18	1.37 ± 0.13
7	0.09 ± 0.099	3.10 ± 0.11	1.11 ± 0.06
8	0.89 ± 0.41	4.93 ± 1.08	22.16 ± 0.83
Etoposide^b^	9.46 ± 0.64	2.98 ± 1.08	31.31 ± 0.76

^a^Other selected ones not listed in the table were inactive (IC_50_ > 40 *μ*M) for cell lines

^b^Etoposide was used as positive controls

**Fig. 7:**
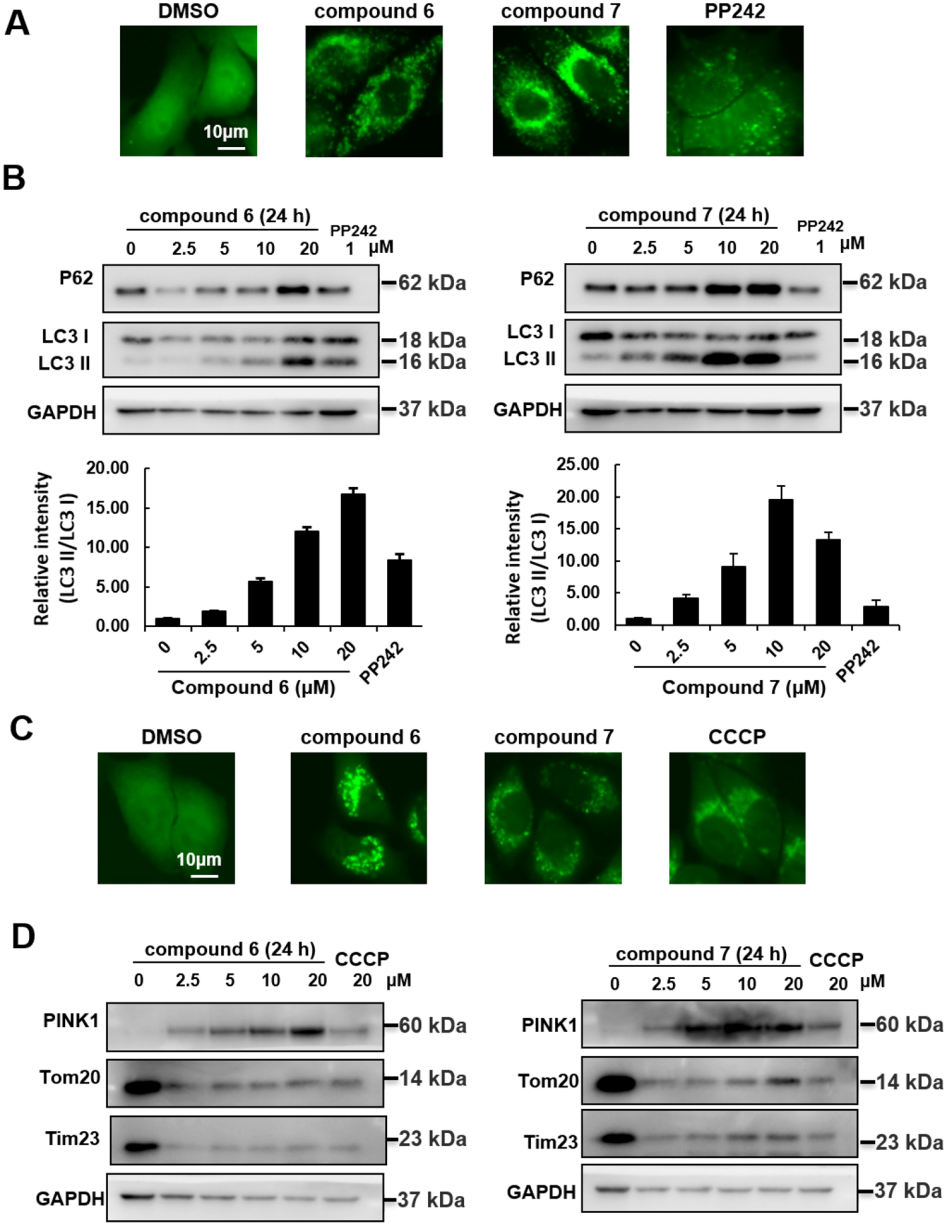
Effects of compounds on autophagy/mitophagy. **a** Induction of autophagy in GFP-LC3 HeLa cells after 24 h and GFP-LC3 puncta were observed with a fluorescent microscope. Scale bars: 10 *μ*M. **b** Autophagy related proteins were analyzed by western blot. **c** Induction of mitophagy in YFP-Parkin HeLa cells after 4 h and YFP-Parkin puncta were observed with a fluorescent microscope. Scale bars: 10 *μ*M. **d** Mitophagy related proteins were analyzed by western blot

**Fig. 8:**
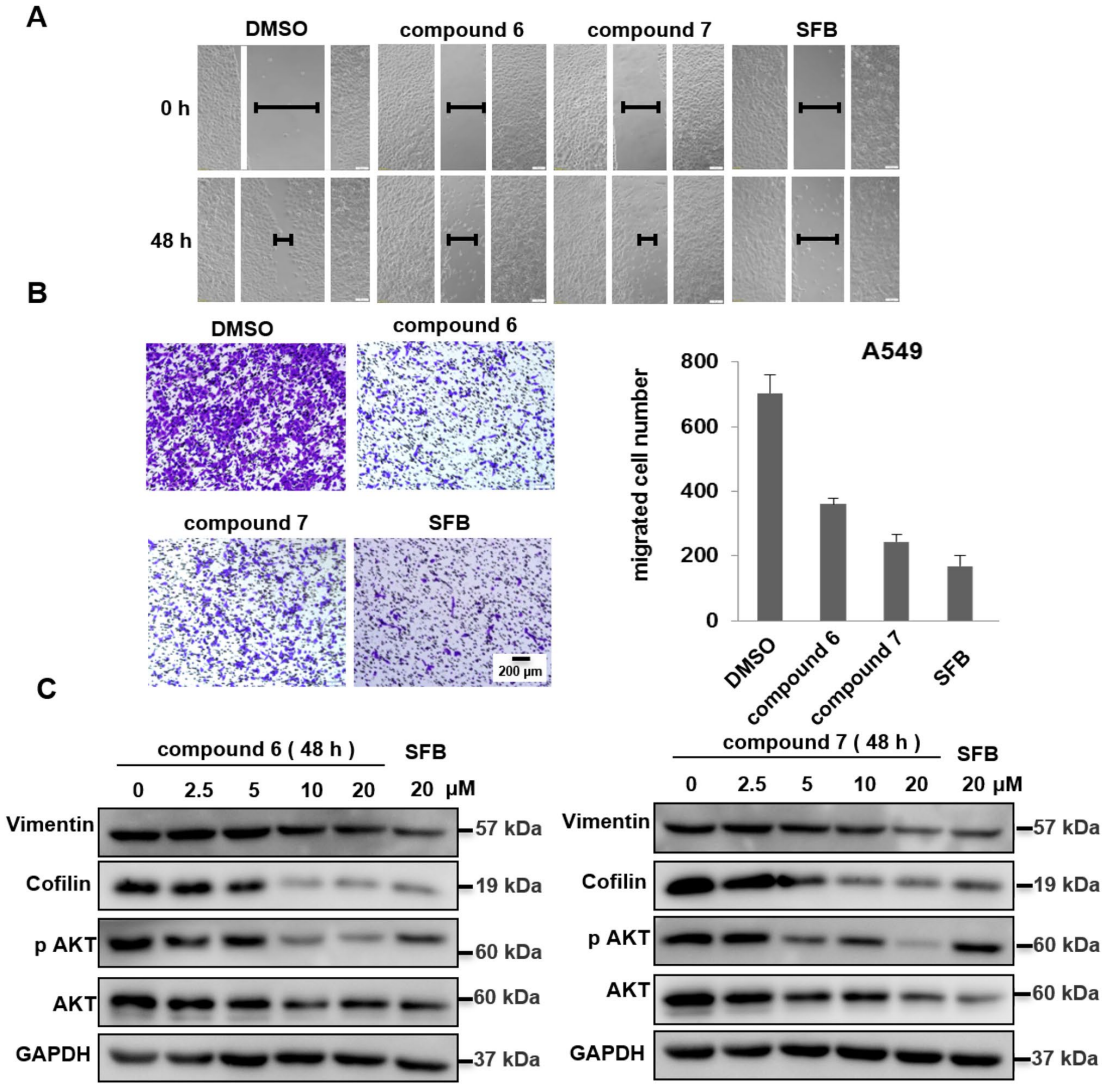
**a** Wound healing assay. A549 cells were treated with compounds **6** and **7** and positive control Sorafenib (20 *μ*M) and monitored with an inverted microscope. **b** Cell migration was measured by transwell assays. The summary data for transwell migration assay was presented as the means ± S.D. Scale bars: 10 *μ*M. **c** A549 cells were treated with compounds **4**–**7** for 24 h and then analyzed by western blot

Autophagy is widely implicated in human diseases, offering a potential target for drug discovery [15]. Then, the effects of **6** and **7** on autophagy were assessed. GFP-LC3 puncta were significantly increased upon these compounds treatment (Fig. 7a). Western blot analysis showed that **6** and **7** inhibited autophagy, as assessed by the increased expression of LC3 II and P62 (Fig. 7b). Similar to CCCP (mitophagy inducer) treatment, **6** and **7** also increased the YFP-Parkin puncta formation (Fig. 7c). These data suggested that the compounds could induce PINK1/Parkin-mediated mitophagy. In addition, the antimetastasis effects of these compounds were also studied. As shown in Fig. 8, wound healing and migration assay suggested **6** and **7** could efficiently suppress cell metastasis consistent with sorafenib (SFB) treated, which also decreased the expression of vimentin, p-AKT and cofilin (Fig. 8). Together, these results indicated that these isolates could suppress lung cancer A549 cells metastasis in vitro and may affect tumor metastasis targeted by Akt and cofilin signaling pathways.

In summary, five new and seven known DIAPs derivatives were isolated from *H. henryi*. Structurally, these compounds were characterized by a dearomatized isoprenylated acylphloroglucinol core combined a functionalized cyclohexane or cyclopentane skeleton. It is worthy to note that several isolates exhibited significant cytotoxic activities in vitro. In addition, they also possess inducing autophagy, mitophagy, and anti-metastasis activities, which provided sufficient information on the potential application of these compounds on future drug development. Therefore, the finding of these DIAPs derivatives with potential antitumor properties may provide a new clue for the discovery of antitumor lead compounds, which should attract great interest from pharmacological and total synthetic communities.

## Experimental

### General Experimental Procedures

Optical rotations were measured on a Jasco P-1020 polarimeter. UV spectra were detected on a Shmadzu UV-2401PC spectrometer. IR spectra were determined on a Bruker FT-IR Tensor-27 infrared spectrophotometer with KBr disks. All 1D and 2D NMR spectra were recorded on Bruker DRX-600 spectrometers using TMS as an internal standard. Unless otherwise specified, chemical shifts (*δ*) were expressed in ppm with reference to the solvent signals. ESIMS and HRESIMS analysis were carried out on Waters Xevo TQS and Aglient G6230 TOF mass spectrometers, respectively. Semi-preparative HPLC was performed on an Aglient 1100 HPLC with a ZORBAX SB-C18 (9.4 × 250 mm) column and a Waters 2695 HPLC with a CHIRALCEL OJ-RH column [4.6 × 150 mm cellulose tris-(4-methylbenzoate) coated on 5 *μ*M silica-gel]. Silica gel (100–200, 200–300 mesh, Qingdao Marine Chemical Co., Ltd., People’s Republic of China), and MCI gel (75–150 *μ*M, Mitsubishi Chemical Corporation, Tokyo, Japan) were used for column chromatography. Fractions were monitored by TLC (GF 254, Qingdao Marine Chemical Co., Ltd.), and spots were visualized by heating silica gel plates sprayed with 10% H_2_SO_4_ in EtOH.

### Plant Material

The plants of *Hypericum henryi* were collected in Dongchuan prefecture (Yunnan Province, People's Republic of China) in September 2018. The plant was identified by ZHANG Yong-Zeng. A voucher specimen (No. 2018H01) was deposited in Kunming Institute of Botany.

### Extraction and Isolation

The sample (20.0 kg) was extracted with MeOH at room temperature and filtered, and the solvent was evaporated in vacuo. The crude extract was subjected to silica gel column chromatography eluted with CHCl_3_ to afford a fraction (695.2 g). This fraction was separated over a MCI-gel column (MeOH-H_2_O from 7:3 to 10:0) to produce five fractions (Fr. A–E). Fr. A (262.3 g) was chromatographed on a silica gel column, eluted with petroleum ether-acetone (100:1 to 0:1), to yield five fractions (Fr. A1–A5). Fr. A2 (37.7 g) was separated over a RP-18 silica column (MeOH–H_2_O from 85:15 to 100:0) and obtained eleven fractions (Fr. A2-1–A2-11). Fr. A2–5 was purified by preparative TLC and semipreparative HPLC to afford **9** (12.3 mg), **10** (11.5 mg) and **2** (10.8 mg). Fr. B (100 g) was chromatographed on a silica gel column, eluted with petroleum ether-ethyl acetate (50:1 to 0:1) to yield ten fractions (Fr. B1–B10). Fr. B3 (11.0 g) was purified by chromatograph on a silica gel column and preparative HPLC (MeOH–H_2_O, 95:5) to afford **11** (25.9 mg) and **12** (4.7 mg). Fr. B4 (755.9 mg) and B6 (1.2 g) were further purified by prearative HPLC (MeOH-H_2_O, 90:10) to afford **1** (15.1 mg), **3** (13.3 mg), **6** (26.5 mg) and **7** (12.0 mg). Fr. B2 (18.0 g) was separated over a RP-18 silica column (MeOH–H_2_O from 85:15 to 100:0), and obtained ten fractions (Fr. B2-1–B2-10) Compounds **4** (7.8 mg), **5** (1.3 mg) and **8** (3.2 mg) were obtained from Fr. B2-2 by preparative HPLC and semipreparative HPLC.

#### Hyperhenol (**1**)

Yellow oil; [*α*] + 250.8 (*c* 0.35, MeOH); UV (MeOH) *λ*_max_ (log *ε*) 202 (4.14), 225 (4.10), 346 (4.02) nm; IR (KBr) *ν*_max_ 3417, 2968, 2932, 1636, 1520, 1460, 1337, 1304, 1233 cm^−1^; ^1^H and ^13^C NMR data, see Tables 1 and 2; ESIMS *m/z* 443 [M − H]^−^; HRESIMS *m/z* 443.2803 [M − H]^−^ (calcd for C_27_H_39_O_5_, 433.2803).

#### Hyperhenol (**2**)

Yellow oil; [*α*] + 53.8 (*c* 0.24, MeOH); UV (MeOH) *λ*_max_ (log *ε*) 203 (4.40), 227 (4.20), 343 (3.90) nm; IR (KBr) *ν*_max_ 3427, 2969, 2927, 1623, 1505, 1448, 1257, 1202 cm^−1^; ^1^H and ^13^C NMR data, see Tables 1 and 2; ESIMS *m/z* 519 [M + H]^+^; HRESIMS *m/z* [M + H]^+^; 519.3106 (calcd for C_33_H_43_O_5_, 519.3105).

#### Hyperhenol (**3**)

Yellow oil; [*α*] + 199.0 (*c* 0.31, MeOH); UV (MeOH) *λ*_max_ (log *ε*) 202 (4.08), 228 (3.98), 239 (3.97), 281 (3.83), 326 (3.91) nm; IR (KBr) *ν*_max_ 3431, 2970, 2932, 2878, 1651, 1522, 1470, 1437 cm^−1^; ^1^H and ^13^C NMR data, see Tables 1 and 2; ESIMS *m/z* 429 [M − H]^−^; HRESIMS *m/z* 429.2653 [M − H]^−^; (calcd for C_26_H_37_O_5_, 429.2646).

#### Hyperhenol (**4**)

Yellow oil; [*α*] + 5.3 (*c* 0.26, MeOH); UV (MeOH) *λ*_max_ (log *ε*) 320 (2.14), 275 (2.30), 241 (2.42), 197 (2.50), 310 (2.17), 269 (2.30), 215 (2.34) nm; IR (KBr) *ν*_max_ 3422, 2970, 2935, 2876, 1657, 1618, 1530, 1462, 1379 cm^−1^; ^1^H and ^13^C NMR data, see Tables 1 and 2; ESIMS *m/z* 427 [M + H]^+^; HRESIMS *m/z* 427.2855 [M + H]^+^ (calcd for C_27_H_38_O_4_, 426.2843).

#### Hyperhenol (**5**)

Yellow oil; [*α*] − 45.0 (*c* 0.12, MeOH); UV (MeOH) *λ*_max_ (log *ε*) 354(2.40), 287(2.17), 231(2.53), 197(2.94), 300(2.15), 276(2.16), 228(2.53), 193(2.81) nm; IR (KBr) *ν*_max_ 3429, 2967, 2926, 2854, 1727, 1659, 1622, 1587, 1512, 1448 cm^−1^; ^1^H and ^13^C NMR data, see Tables 1 and 2; ESIMS *m/z* 501 [M + H]^+^; HRESIMS *m/z* 501.3008 [M + H]^+^ (calcd for C_33_H_40_O_4_, 501.2999).

#### X-ray Crystallographic Analysis of Hyperhenone E (**8**)

C_26_H_38_O_5_, *M* = 430.56, *a* = 22.6496(4) Å, *b* = 9.4550(2) Å, *c* = 23.8898(4) Å, *α* = 90°, *β* = 94.6410(10)°, *γ* = 90°, *V* = 5099.27(16) Å^3^, *T* = 100(2) K, space group *P*21, *Z* = 8, *μ* (CuK*α*) = 0.609 mm^−1^, 56,500 reflections measured, 17,671 independent reflections (R_int_ = 0.0269). The final *R*_*1*_ values were 0.0363 (*I* > 2*σ*(*I*)). The final *wR*(*F*^2^) values were 0.0966 (*I* > 2*σ*(*I*)). The final *R*_1_ values were 0.0366 (all data). The final *wR*(*F*^2^) values were 0.0971 (all data). The goodness of fit on *F*^2^ was 1.056. Flack parameter = 0.02(2). Crystallographic data for the structure of **8** have been deposited in the Cambridge Crystallographic Data Centre (deposition number: CDCC 1,941,889).

#### Cell Culture

HeLa cells, GFP-LC3 HeLa cells, YFP-Parkin HeLa cells and A549 cells were maintained in DMEM (Gibco, D11527) supplemented with 10% fetal bovine serum, FBS (HyClone, SV30160.03) and 100 U/mL penicillin–streptomycin (Gibco/Invitrogen, 15,140–122) in a humidified atmosphere containing 5% CO_2_ at 37 °C.

##### MTT Assay and Determination of IC_50_

The cells were seeded in a 96-well tissue culture plate at a predetermined density in 100 *μ*L of complete medium, attached overnight, and then treated with a series of concentrations of compound for 72 h. At the end of the incubation period, 10 *μ*L MTT solution was added into each well of a 96-well plate for 4 h at 37℃. After the medium was removed, 100 *μ*L DMSO was added to dissolve the purple crystals. After shaking for 5 min, the optical densities at 490 nm were measured using a Microplate Reader.

##### MitoTracker Red Staining

HeLa cells were seeded on coverslips and treated with compounds **6** and **7** for 48 h. We then removed the media from the dish and added staining solution containing MitoTracker red (100 nM) incubation 30 min at 37 °C. The cells were fixed with 4% PFA in PBS for 15 min and observed using a fluorescence microscope.

##### Flow Cytometry Analysis

HeLa cells were treated with various concentrations of **7** and **8** for 48 h. Subsequently, the cells were harvested, washed with PBS and fixed with 70% alcohol at 4 °C overnight. Then cells were washed with PBS and stained with 20 *μ*g/mL PI/RNase staining buffer for 30 min and analyzed using FACSCalibur flow cytometer (Becton Dickinson, USA).

#### Immunofluorescence Microscopy

The GFP-LC3 or YFP-Parkin HeLa cells were treated with compounds for the indicated time point, and then the cells were fixed with 4% PFA in PBS for 15 min at room temperature. The cells were observed under a fluorescence microscope (Olympus, IX83).

#### Wound Healing Assay

Wound healing was used to evaluate cell motility as our previous study [16]. Briefly, A549 cells were seeded into a 24-well culture plate. When the cells grew to 90% confluence, then a scratch was gently created through the cell monolayer by sterile 10 *μ*L pipette tips and loose cells were washed away. The cell migration was observed and imaged under an IX83 microscope for each condition and timepoint (0, 48 h). (Olympus, Tokyo, Japan).

#### Cell Migration Assay

Cell migration assay were performed as described previously [17]. In brief, cell migration was estimated using transwell chambers (Millicell, Germany) with a pore size of 8 *μ*M. For the migration assay, 4.5 × 104 A549 cells resuspended in 100 *μ*L serum-free medium were seeded in the upper chamber with serum-containing medium in the lower chamber of 24-well transwell plates (BD Biosciences, San Jose, CA). After 24 h, the experiment was terminated by wiping the cells from the wells with a cotton swab and fixed and stained with 0.05% crystal violet for 20 min, scored under a light microscope in five random fields.

#### Western Blotting Analysis

Cells were harvested and lysed in a lysis buffer (62.5 mM Tris at pH 6.8, 20% glycerol, 2% SDS, phosphatase inhibitor), proteins were separated on SDS polyacrylamide gels and transferred to PVDF membranes (Millipore, Billerica, MA, USA). The membranes were blocked with 5% nonfat milk, and immunoblotted with primary antibodies at 4 °C overnight. After washed three times with TBST, membranes were incubated for 1 h with appropriate secondary antibodies at room temperature. The follow antibodies were used in our experiments: Caspase-3 (CST, 9662), Cleaved-caspase-3 (CST, 9661), Caspase-9 (CST, 9502), PARP (CST, 9542), LC3 (Sigma, L7543), P62 (BML, PM045), PINK1 (CST, 6946), Tim23 (BD, 611222), Tom20 (sc-17764), E-cadherin (CST, 3195), Vimentin (CST, 5741), pAKT (Ser473, CST, 9171), AKT (CST, 9272), Cofilin (CST, 5175) and GAPDH (CST, 5174). GAPDH was used as the loading control. Membranes were visualized with Image Quant LAS 4000 (General Electric Company).

## Electronic supplementary material

Below is the link to the electronic supplementary material.

Supplementary file1 (DOC 6653 kb)
