# Supplementary material for: Dearomatized Isoprenylated Acylphloroglucinol Derivatives with Potential Antitumor Activities from Hypericum henryi
Source: Nat Prod Bioprospect. 2020 Feb 4;10(1):1–11. doi: 10.1007/s13659-019-00229-w (PMC7046846; doi:10.1007/s13659-019-00229-w)
Supplement: Supplementary file 1 — Supplementary file1 (DOC 6653 kb) [file 13659_2019_229_MOESM1_ESM.doc]

**Dearomatized isoprenylated acylphloroglucinol derivatives with potential antitumor activities from *Hypericum henryi***

Yan-Song Ye^1,4^, Man Wu^2^, Na-Na Jiang^1,4^, Yuan-Zhi Lao^2^, Wen-Wei Fu^2^, Xia Liu^3^, Xing-Wei Yang^1^, Juan Zhang^2^, Hong-Xi Xu^2*^, Gang Xu^1*^

*^1^ State Key Laboratory of Phytochemistry and Plant Resources in West China and Yunnan Key Laboratory of Natural Medicinal Chemistry, Kunming Institute of Botany, Chinese Academy of Sciences, Kunming 650201, China*

*^2^ School of Pharmacy, Shanghai University of Traditional Chinese Medicine, Shanghai, 201203, China*

*^3^ Department of Pharmacy, Chongqing Traditional Chinese Medicine Hospital, Chongqing 400021, China*

*^4^* *University of Chinese Academy of Sciences, Beijing 100049, China*

Yan-Song Ye, Man Wu have contributed equally to this work.

** Corresponding author.**^E-mail address^:* ^Hong-Xi Xu^: [xuhongxi88@gmail.com](mailto:xuhongxi88@gmail.com)^, Gang Xu^: [xugang008@mail.kib.ac.cn](mailto:xugang008@mail.kib.ac.cn)

Supplementary data

**Figure S1–S8.**The original NMR and MS spectra compound **1**

**Figure S9–S16.**The original NMR and MS spectra compound **2**

**Figure S17–S24.**The original NMR and MS spectra compound **3**

**Figure S25–S32.**The original NMR and MS spectra compound **4**

**Figure S33–S40.**The original NMR and MS spectra compound **5**


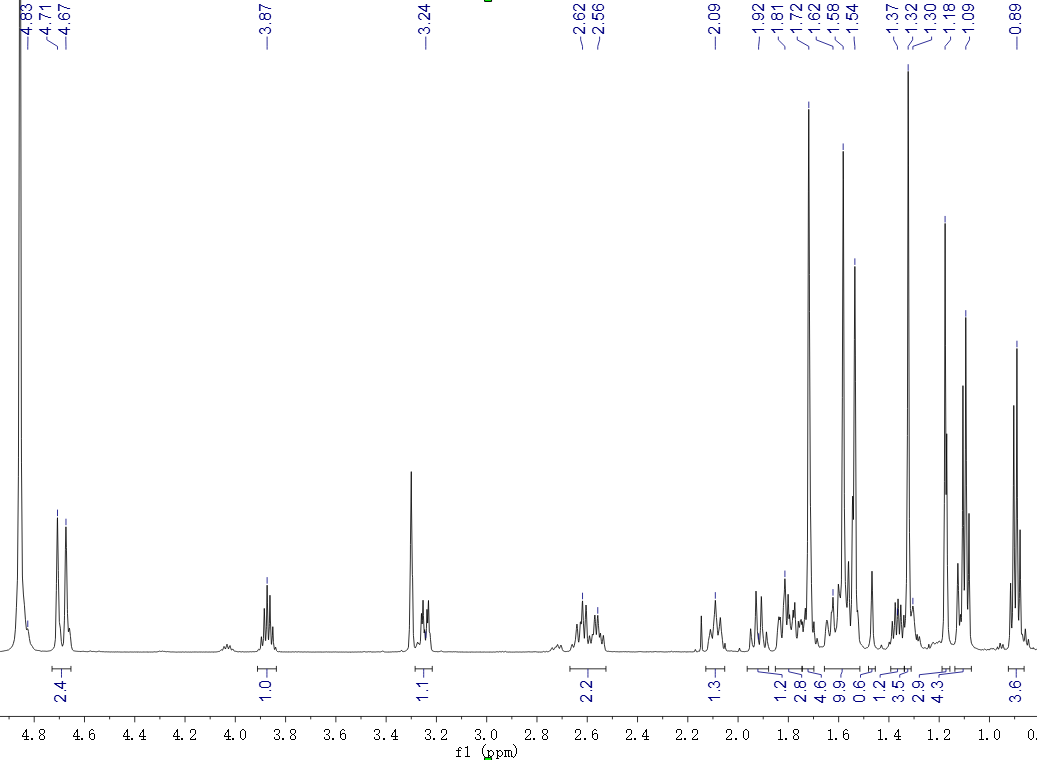

**Fig. S1** ^1^H NMR spectrum of hyperhenol A (**1**) in CD_3_OD


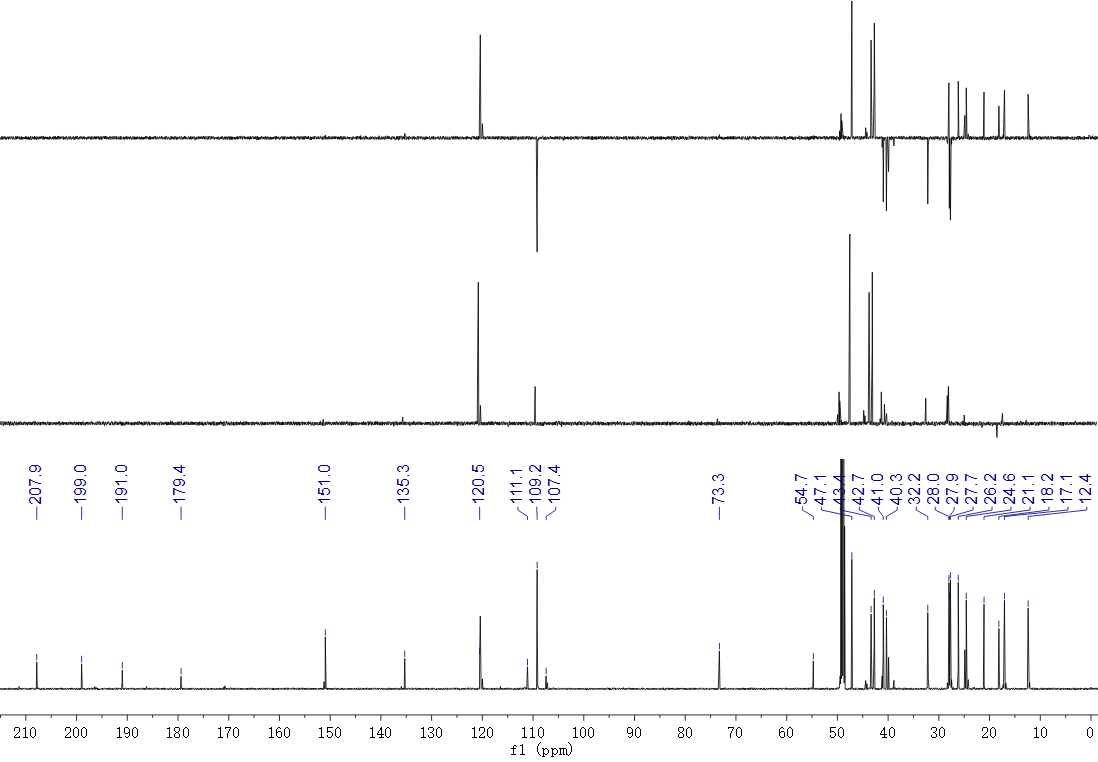


**Fig. S2** ^13^C and DEPT NMR spectrum of hyperhenol A (**1**) in CD_3_OD


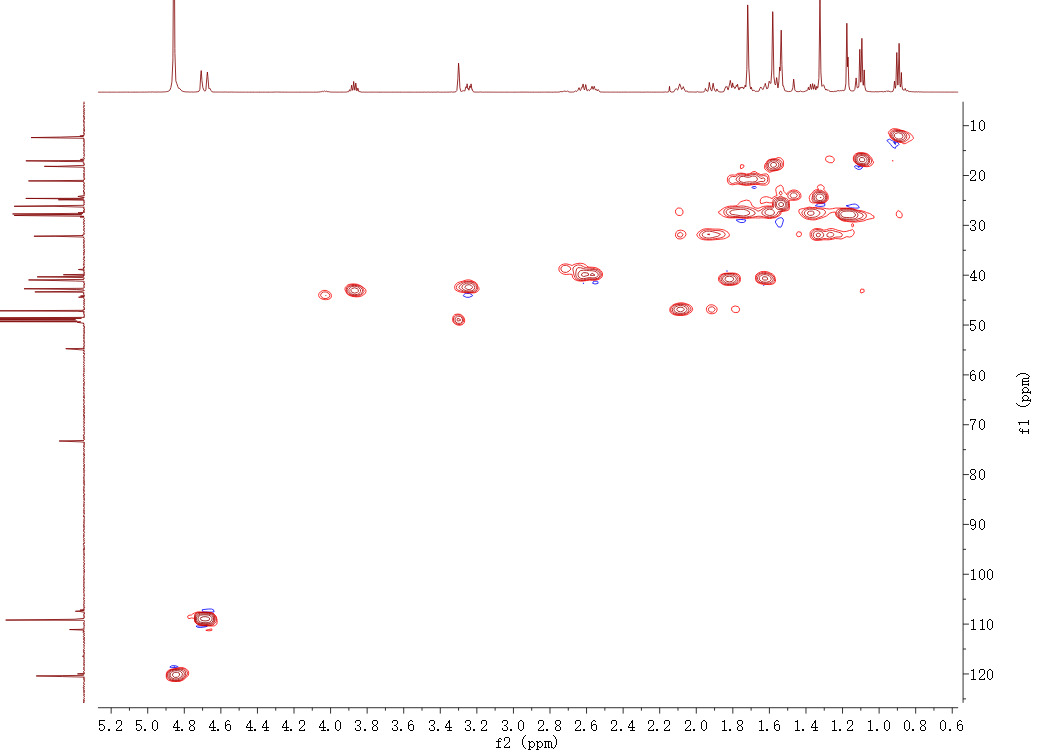

**Fig. S3** HSQC spectrum of hyperhenol A (**1**) in CD_3_OD


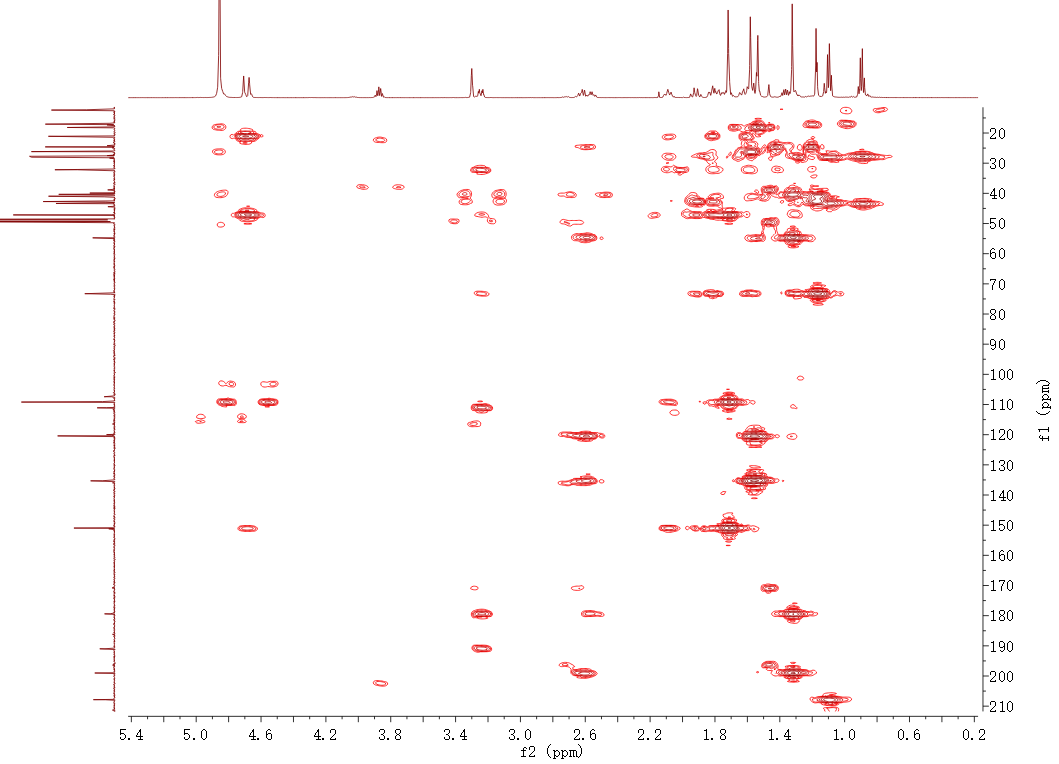

**Fig. S4** HMBC spectrum of hyperhenol A (**1**) in CD_3_OD


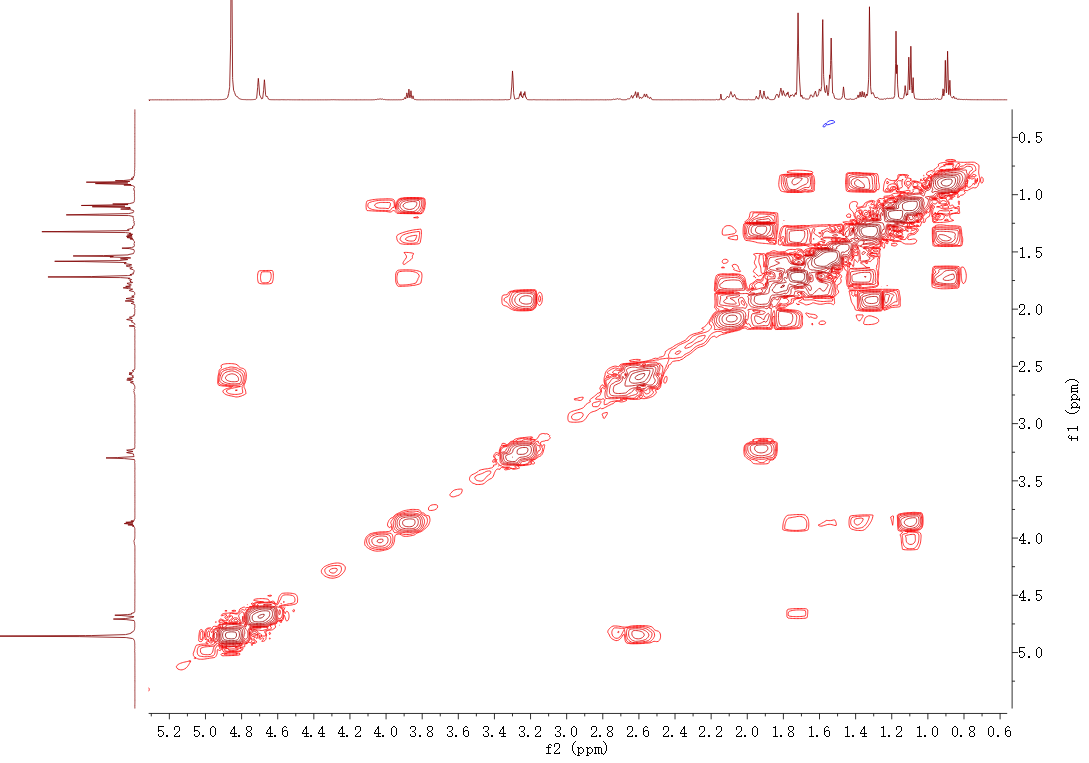

**Fig. S5** ^1^H-^1^H COSY spectrum of hyperhenol A (**1**) in CD_3_OD
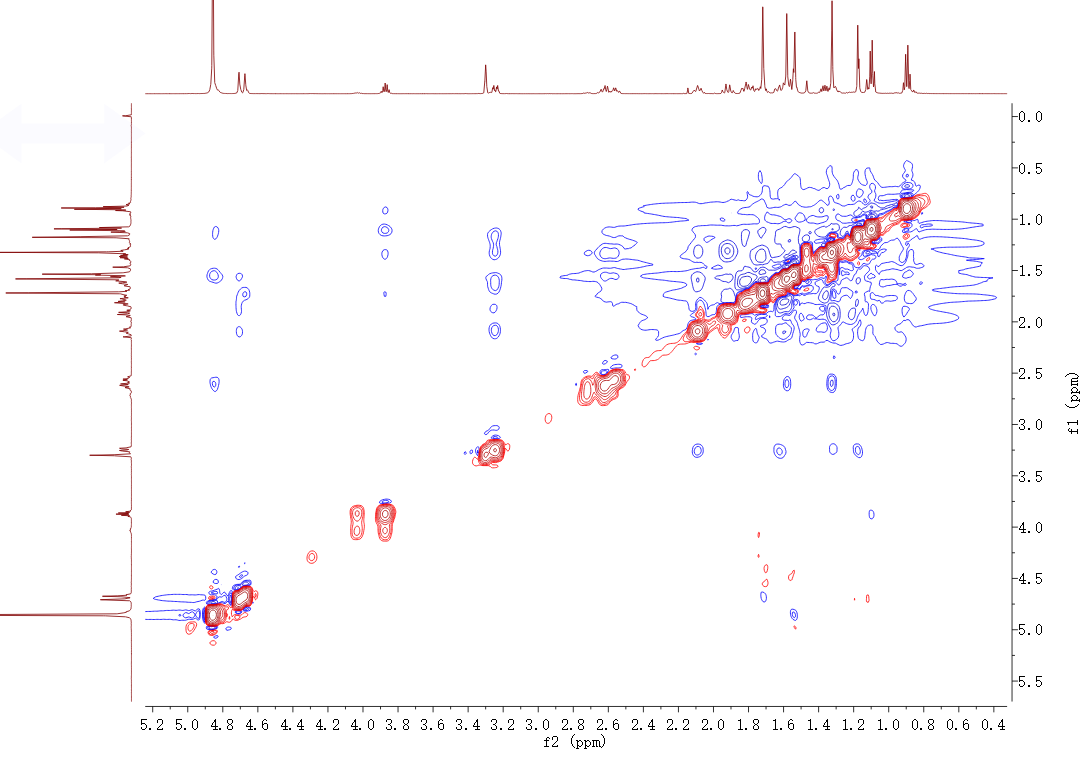


**Fig. S6** ROESY spectrum of hyperhenol A (**1**) in CD_3_OD


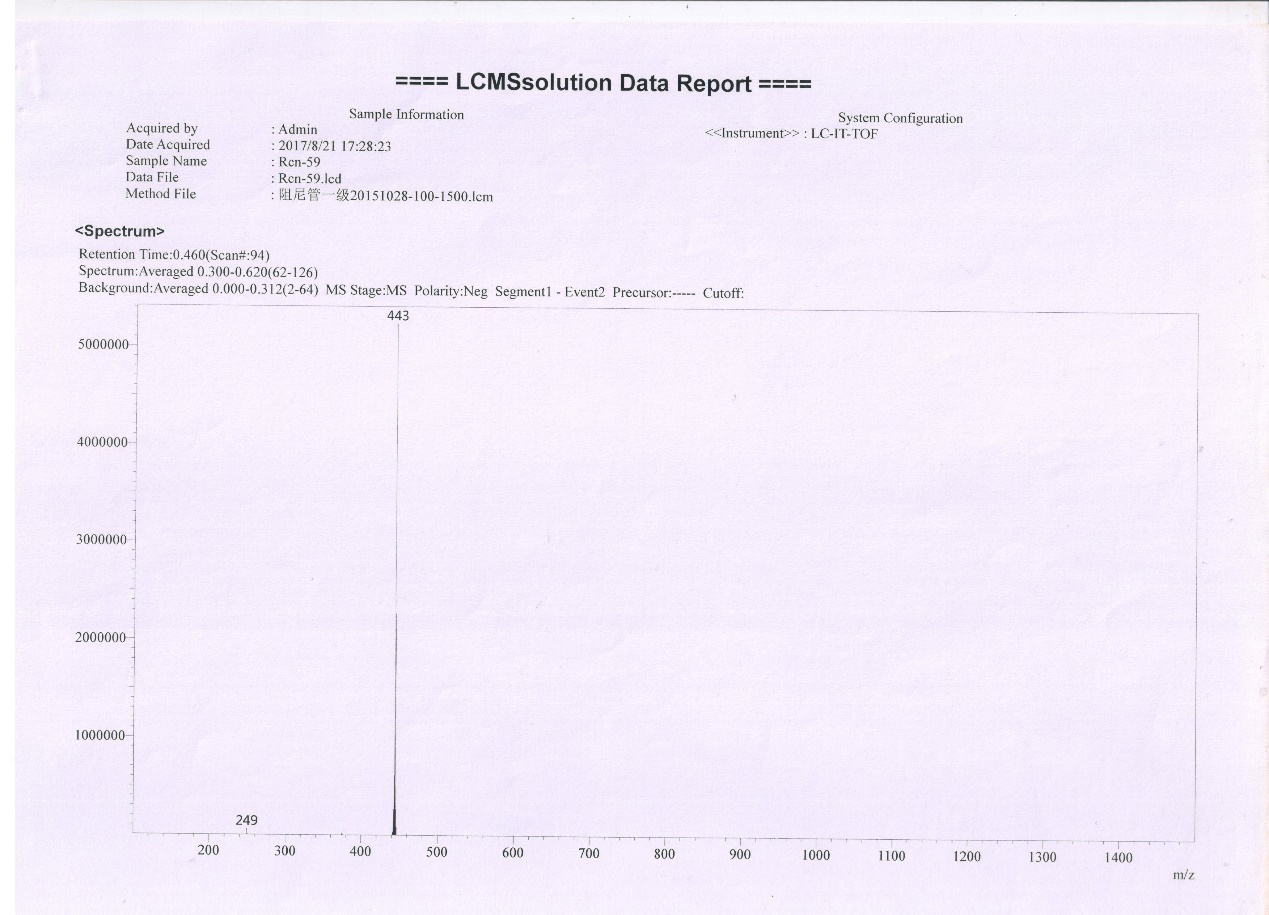

**Fig. S7** ESIMS spectroscopic report of hyperhenol A (**1**)


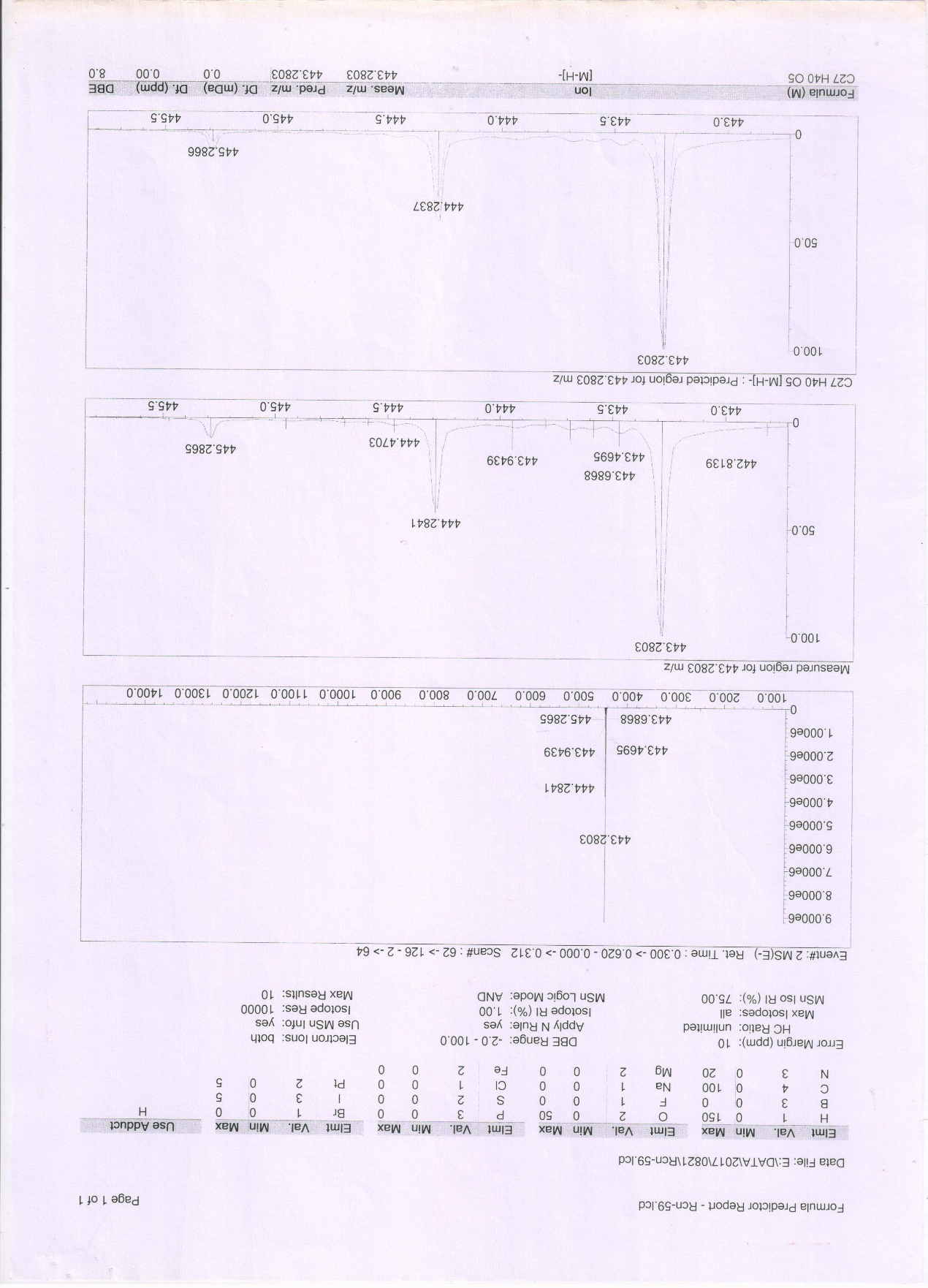

**Fig. S8** HRESIMS spectroscopic report of hyperhenol A (**1**)


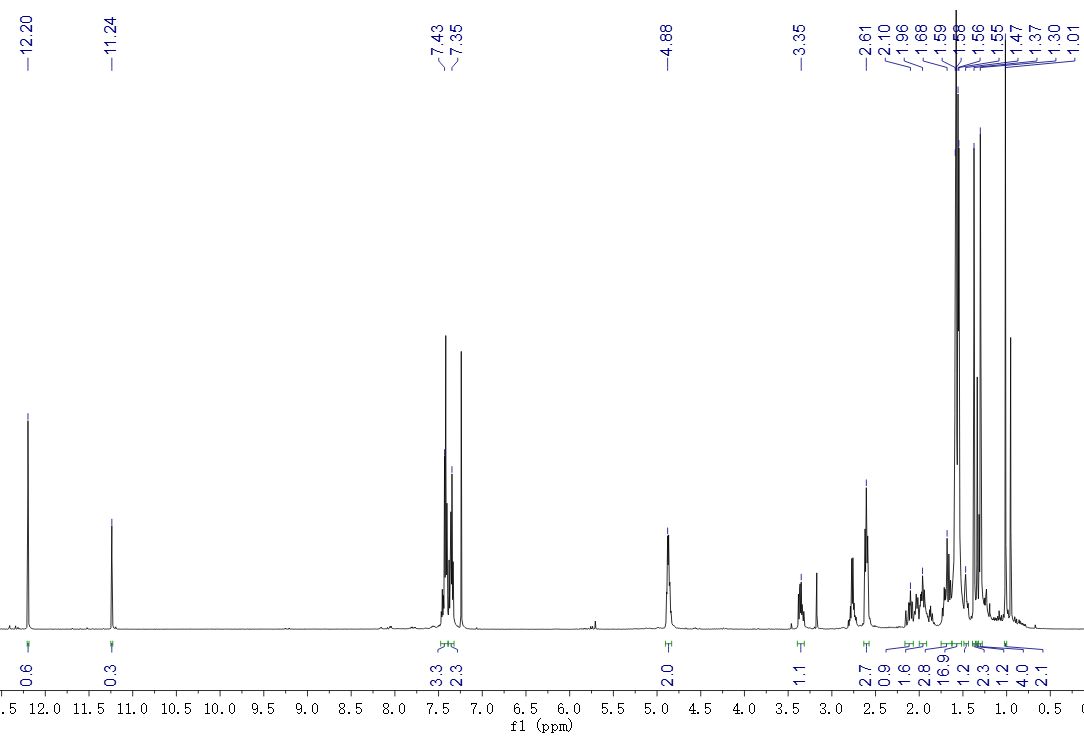


**Fig. S9** ^1^H-NMR spectrum of hyperhenol B (**2**) in CDCl_3_


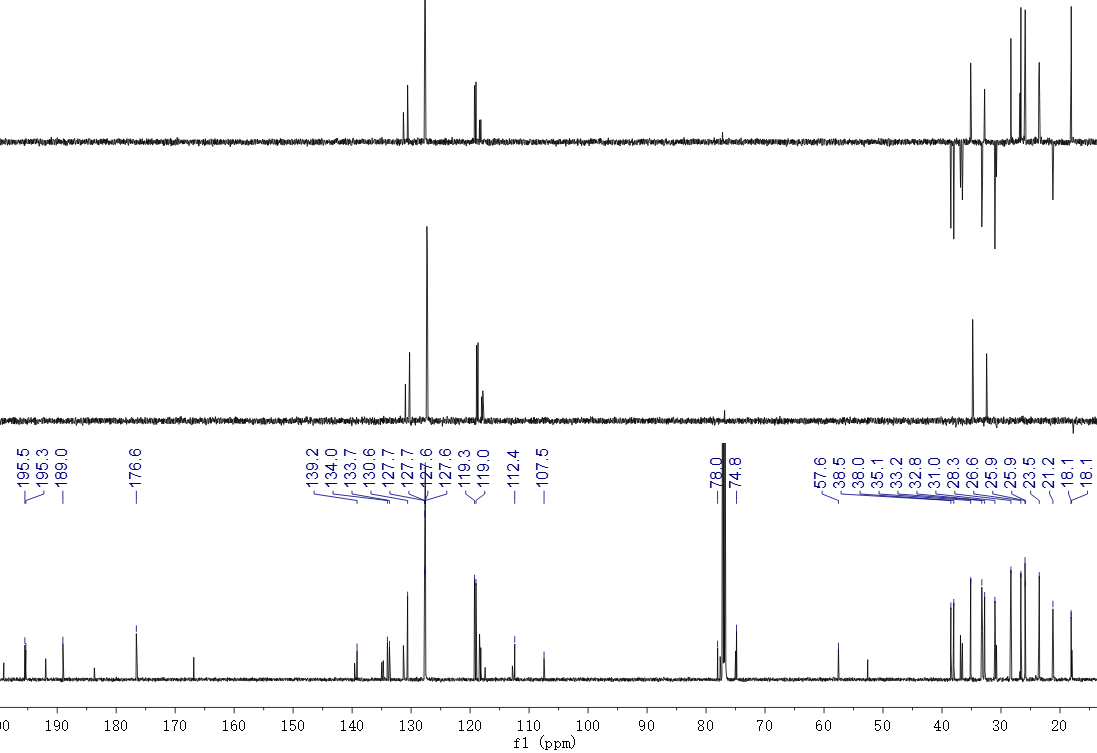


**Fig. S10** ^13^C-NMR spectrum of hyperhenol B (**2**) in CDCl_3_


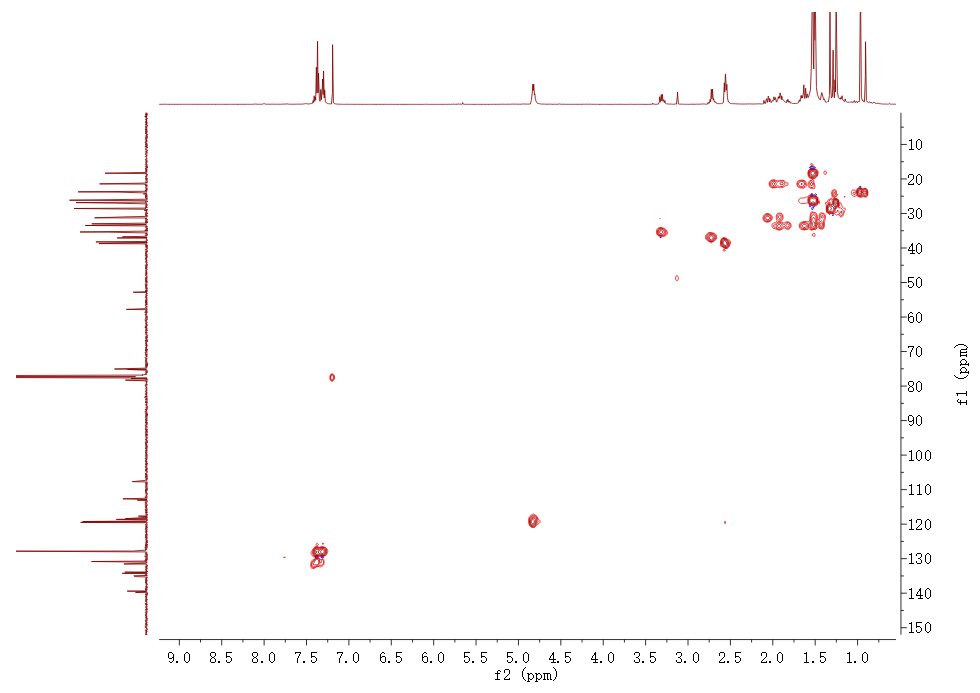


**Fig. S11** HSQC spectrum of hyperhenol B (**2**) in CDCl_3_


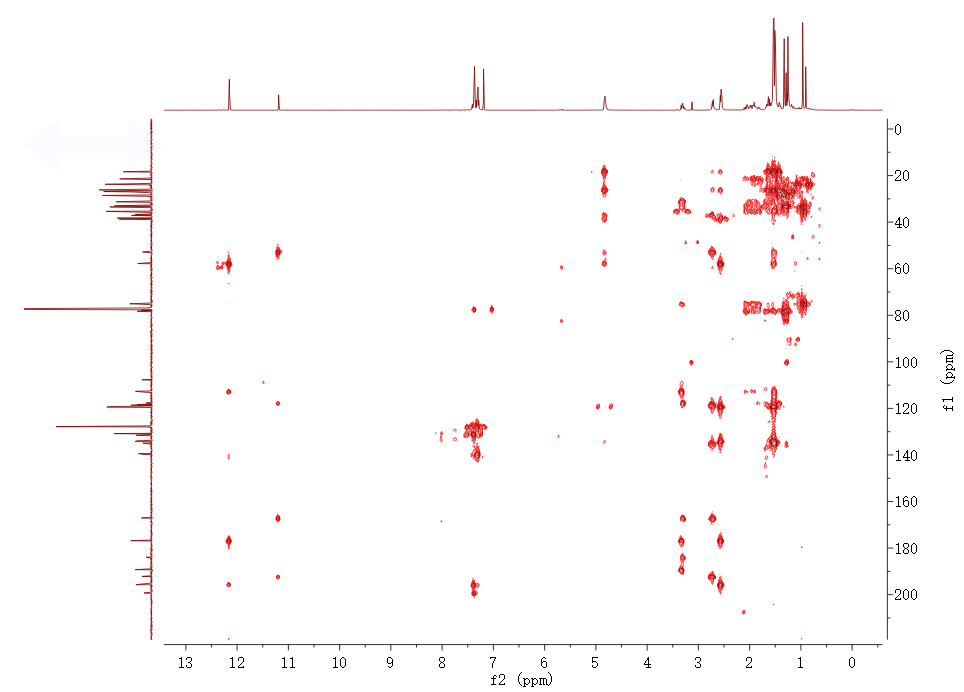


**Fig. S12** HMBC spectrum of hyperhenol B (**2**) in CDCl_3_


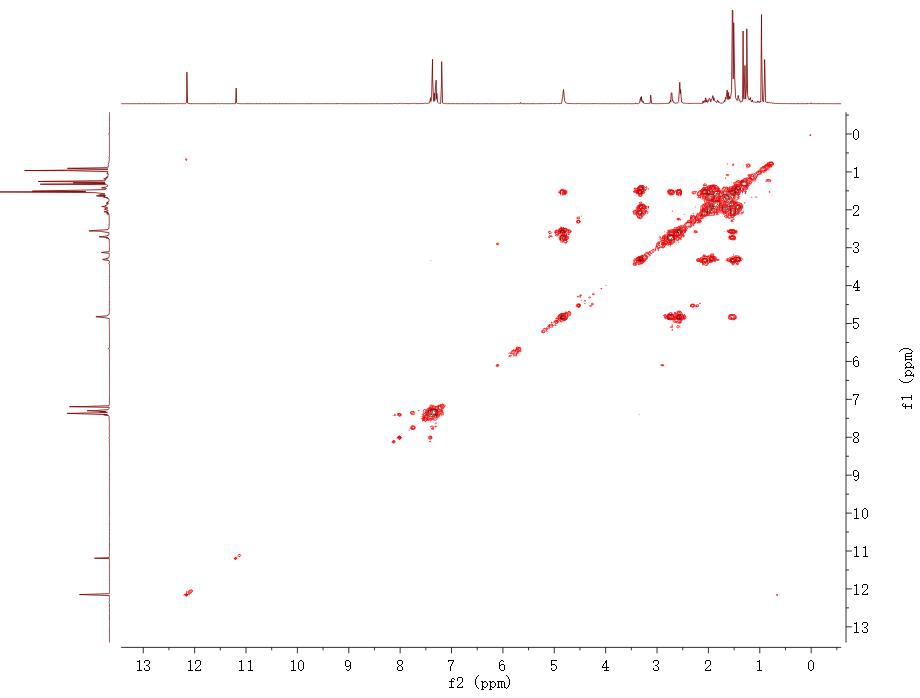


**Fig. S13** ^1^H-^1^H COSY spectrum of hyperhenol B (**2**) in CDCl_3_


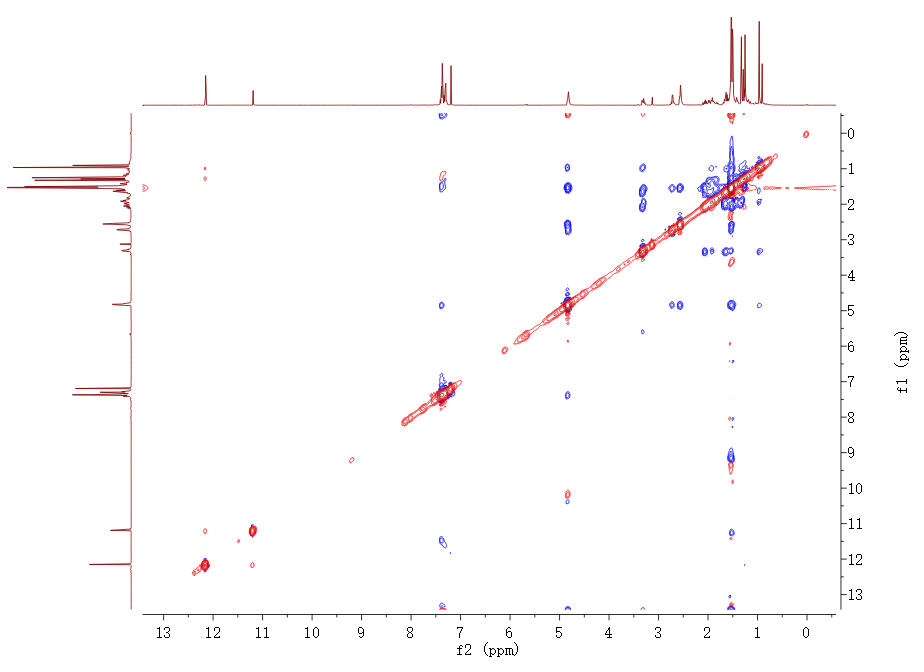


**Fig. S14** ROESY spectrum of hyperhenol B (**2**) in CDCl_3_


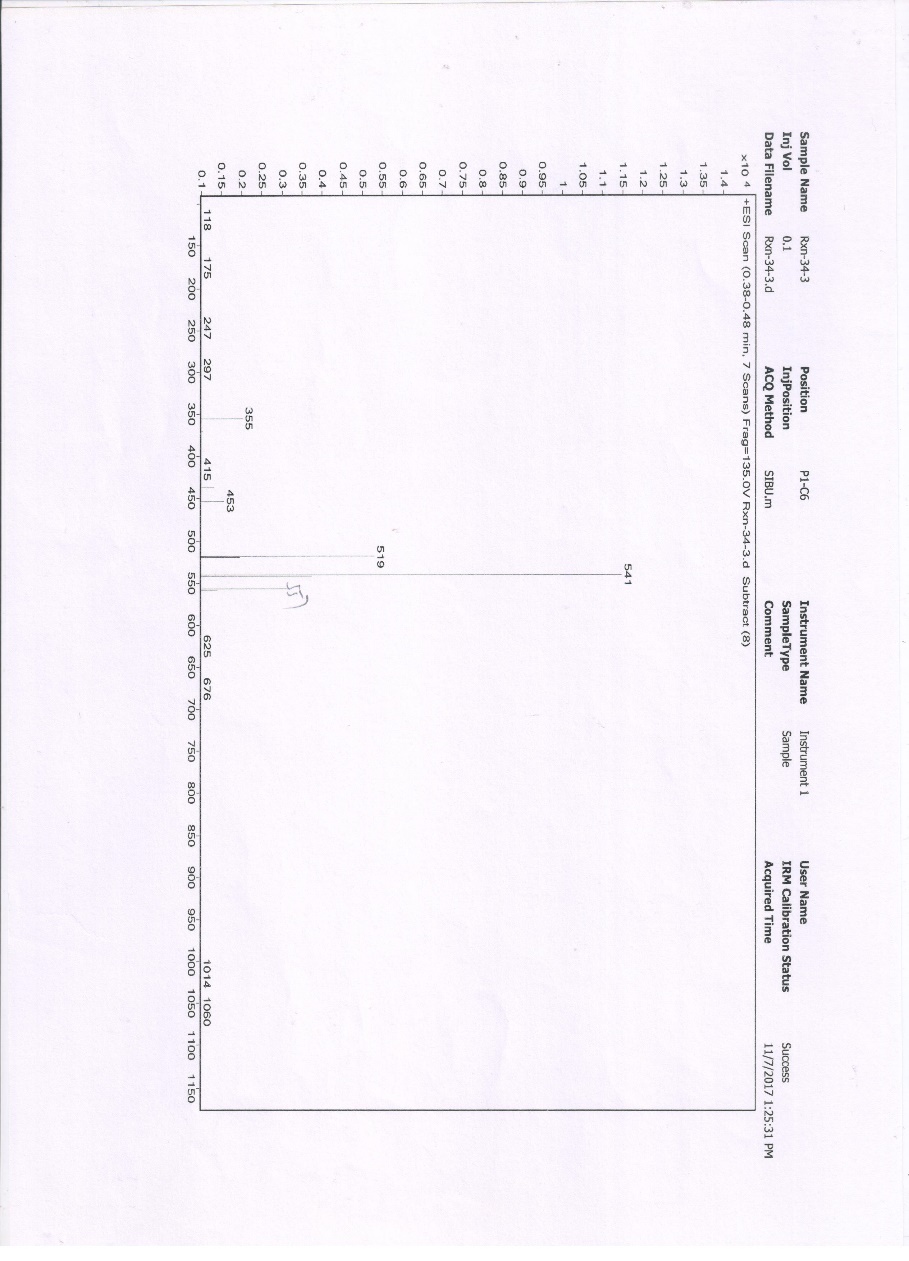


**Fig. S15** ESIMS spectroscopic report of hyperhenol B (**2**)


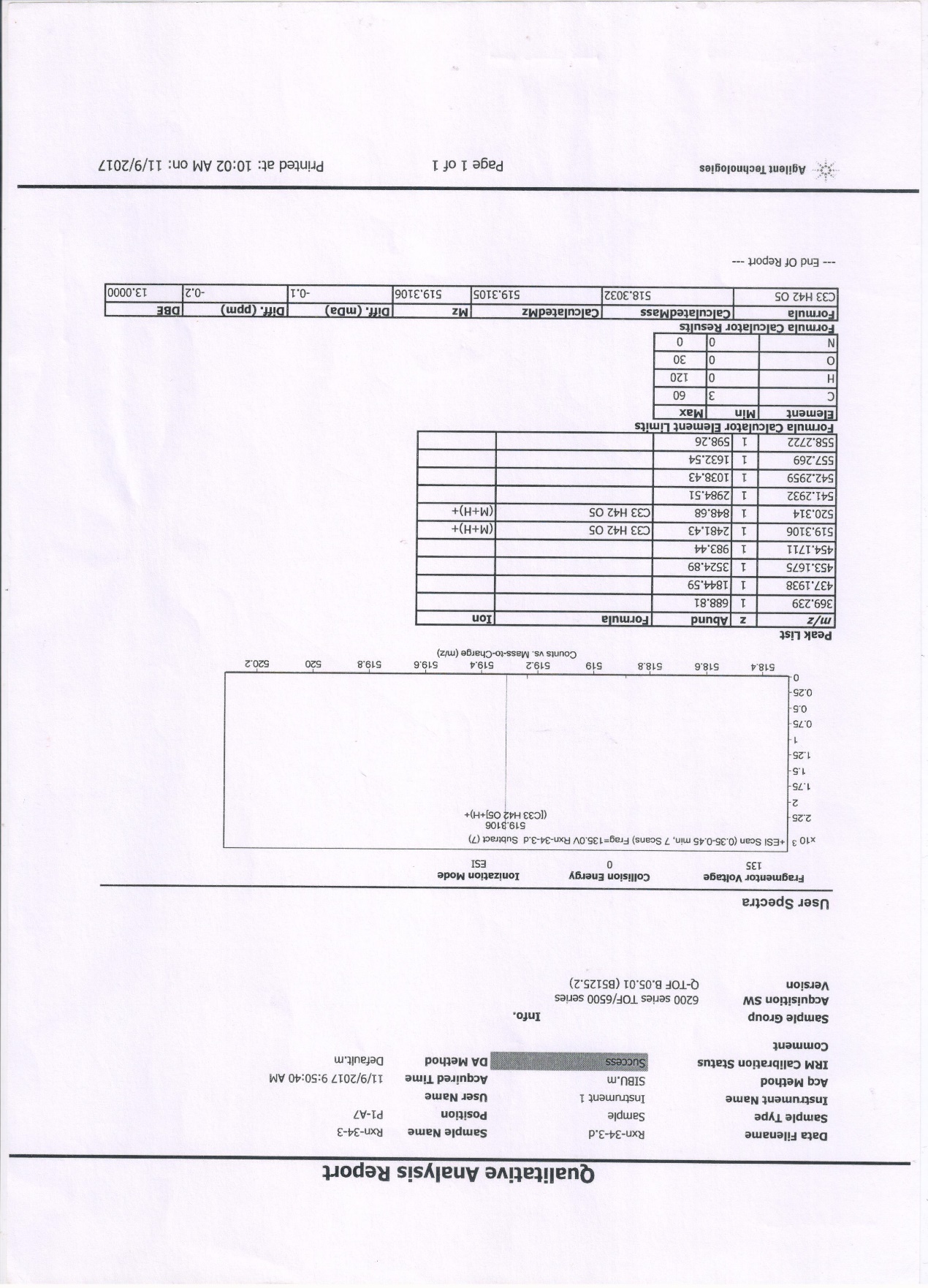


**Fig. S16** HRESIMS spectroscopic report of hyperhenol B (**2**)


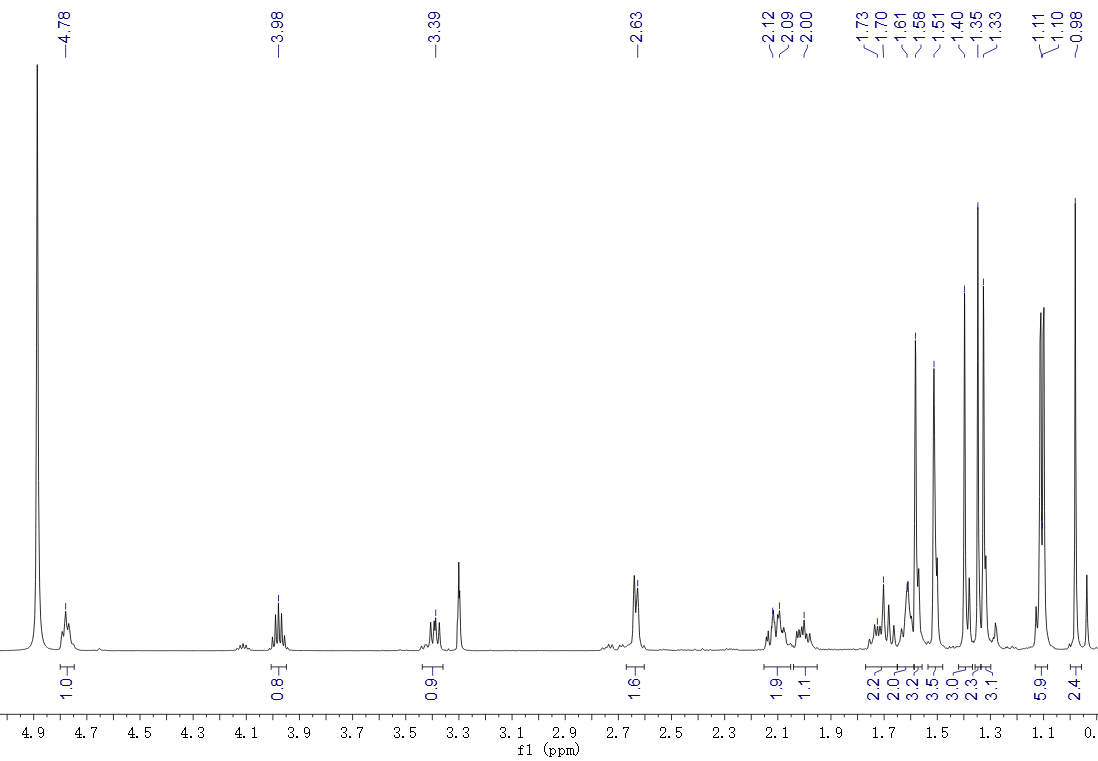


**Fig. S17** ^1^H NMR spectrum of hyperhenol C (**3**) in CD_3_OD


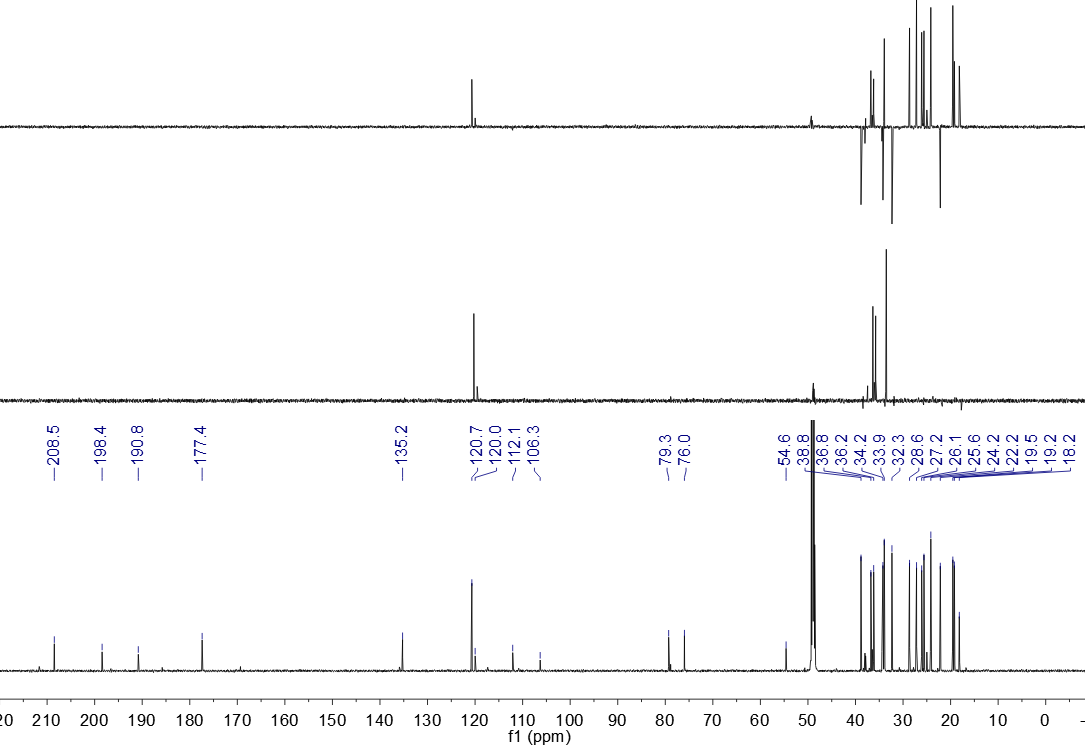


**Fig. S18** ^13^C NMR spectrum of hyperhenol C (**3**) in CD_3_OD


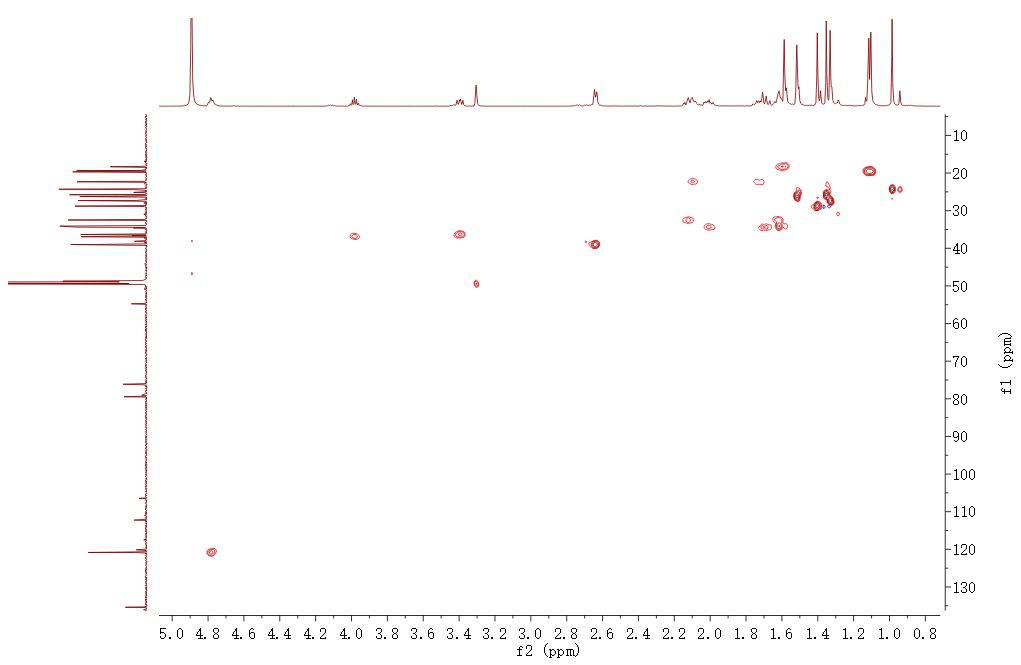


**Fig. S19** HSQC spectrum of hyperhenol C (**3**) in CD_3_OD


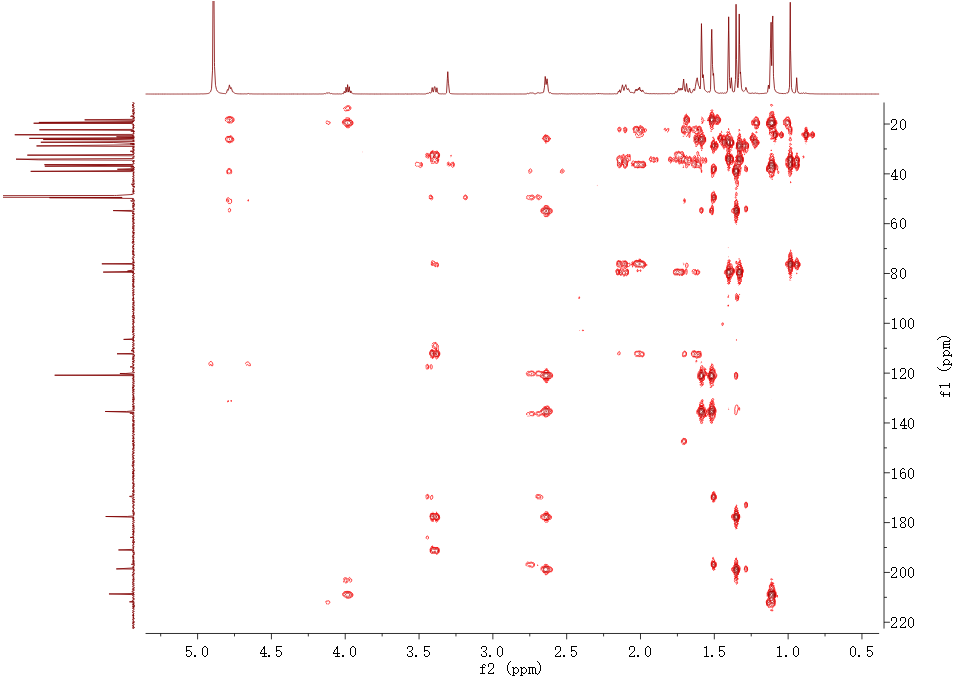


**Fig. S20** HMBC spectrum of hyperhenol C (**3**) in CD_3_OD


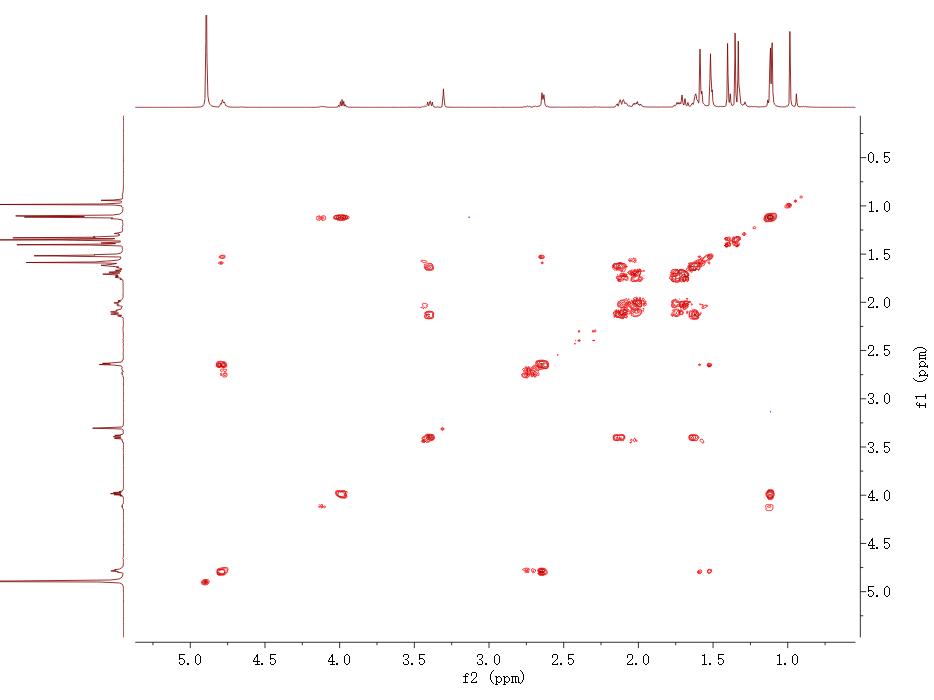


**Fig. S21** ^1^H-^1^H COSY spectrum of hyperhenol C (**3**) in CD_3_OD


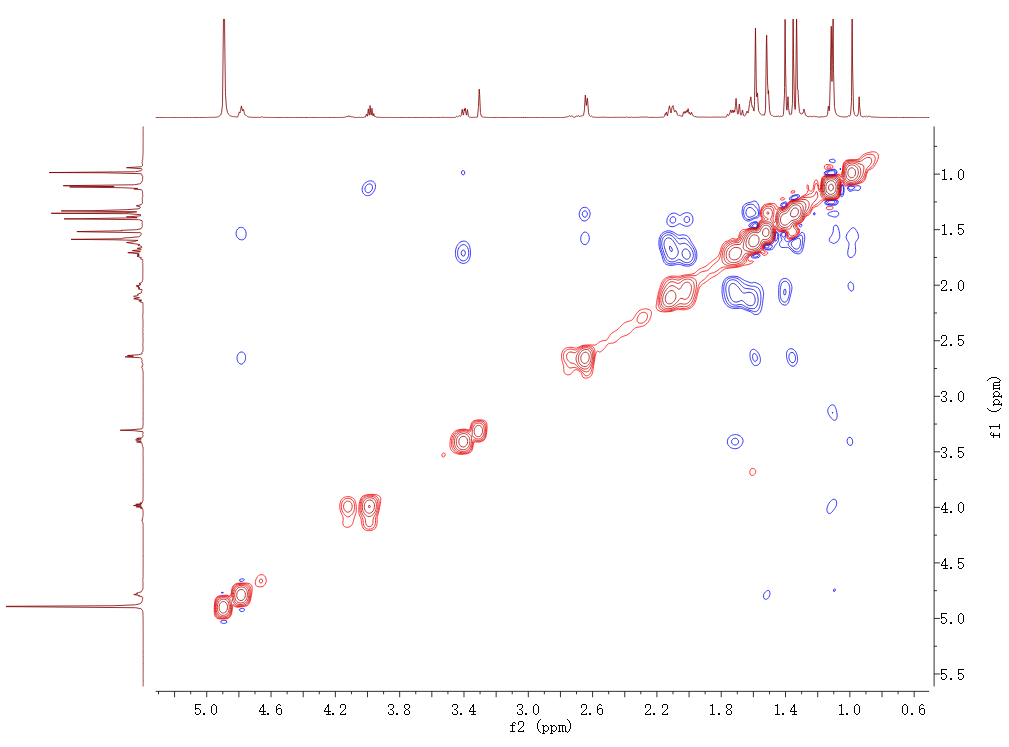


**Fig. S22** ROESY spectrum of hyperhenol C (**3**) in CD_3_OD


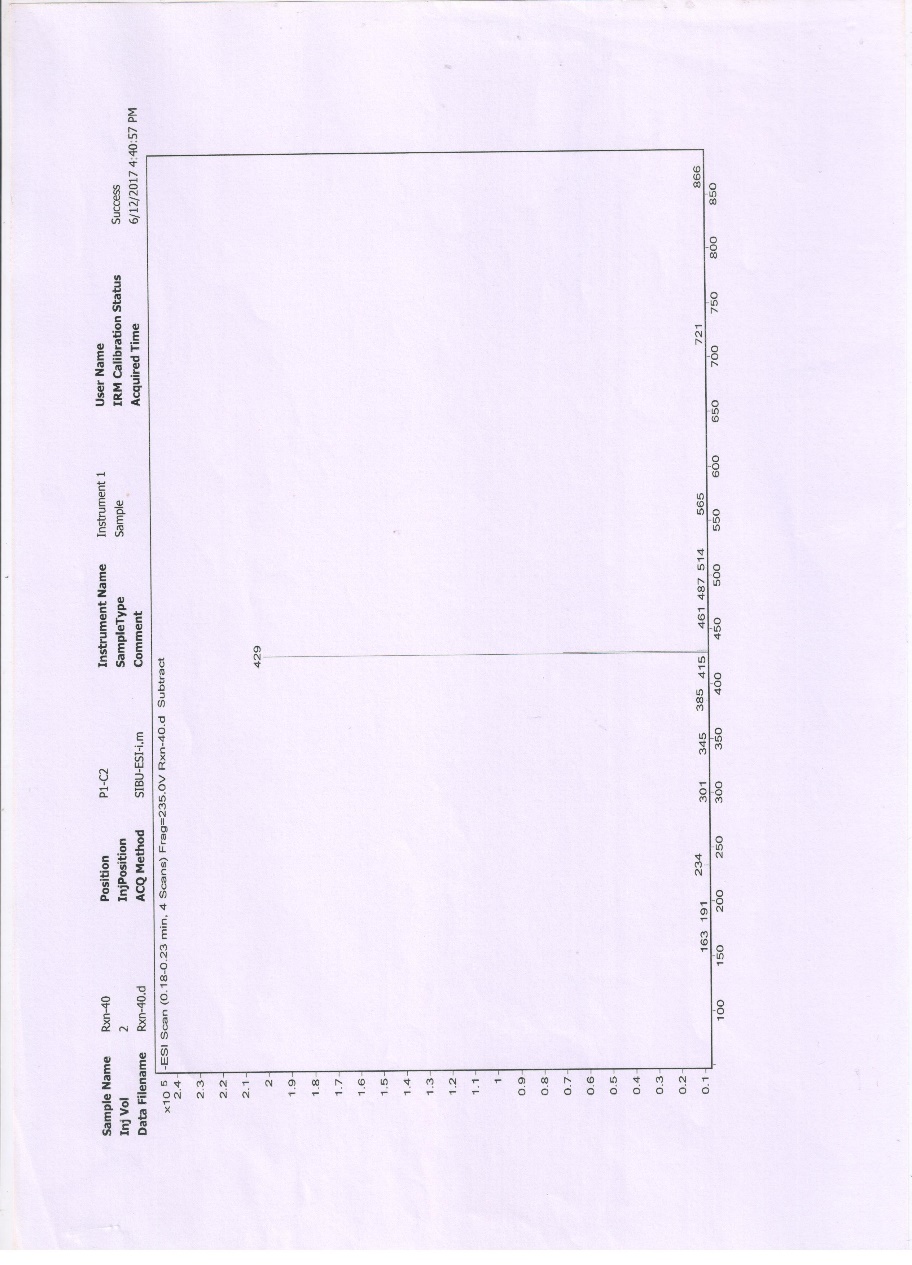


**Fig. S23** ESIMS spectroscopic report of hyperhenol C (**3**)


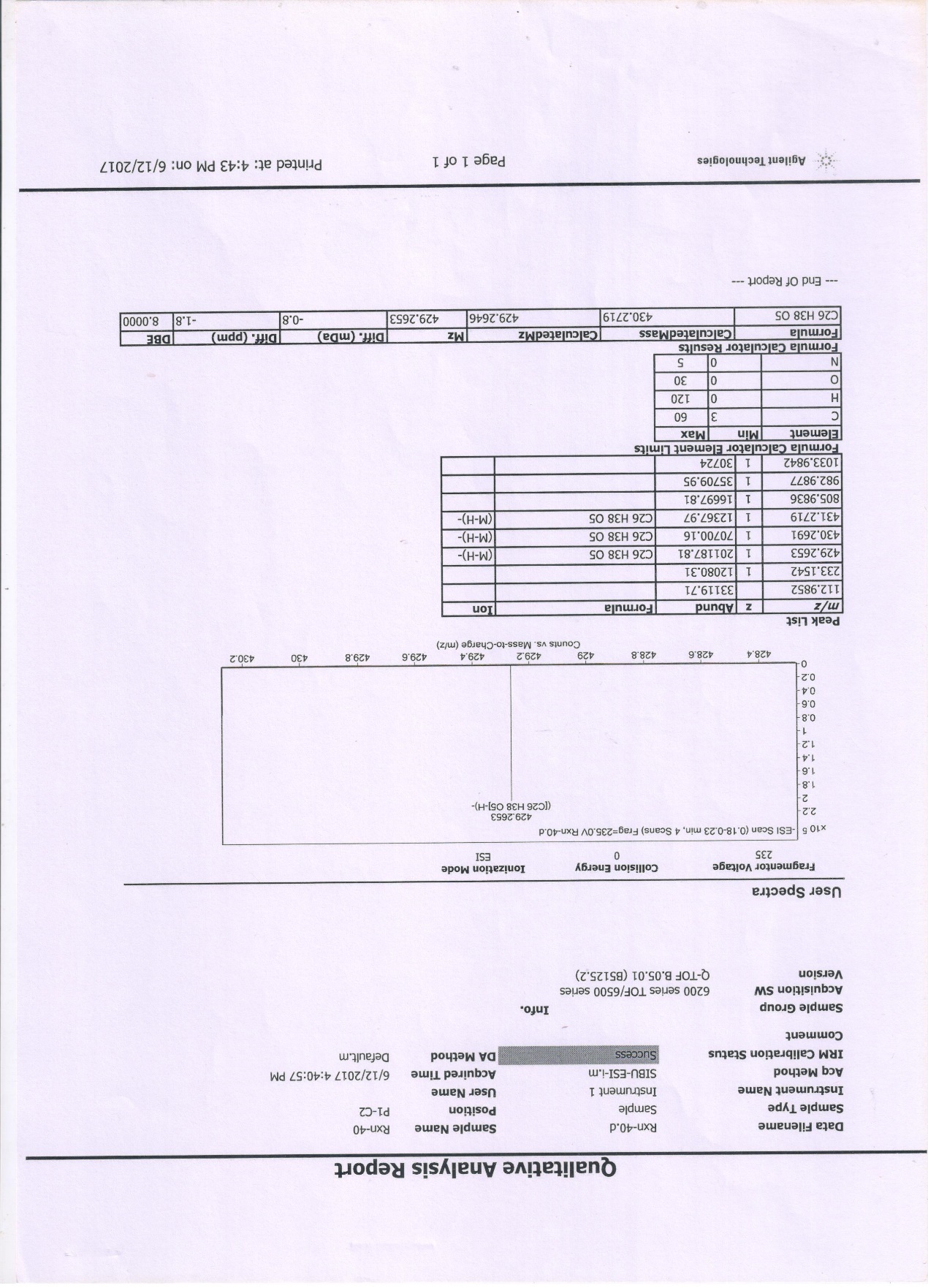


**Fig. S24** HRESIMS spectroscopic report of hyperhenol C (**3**)


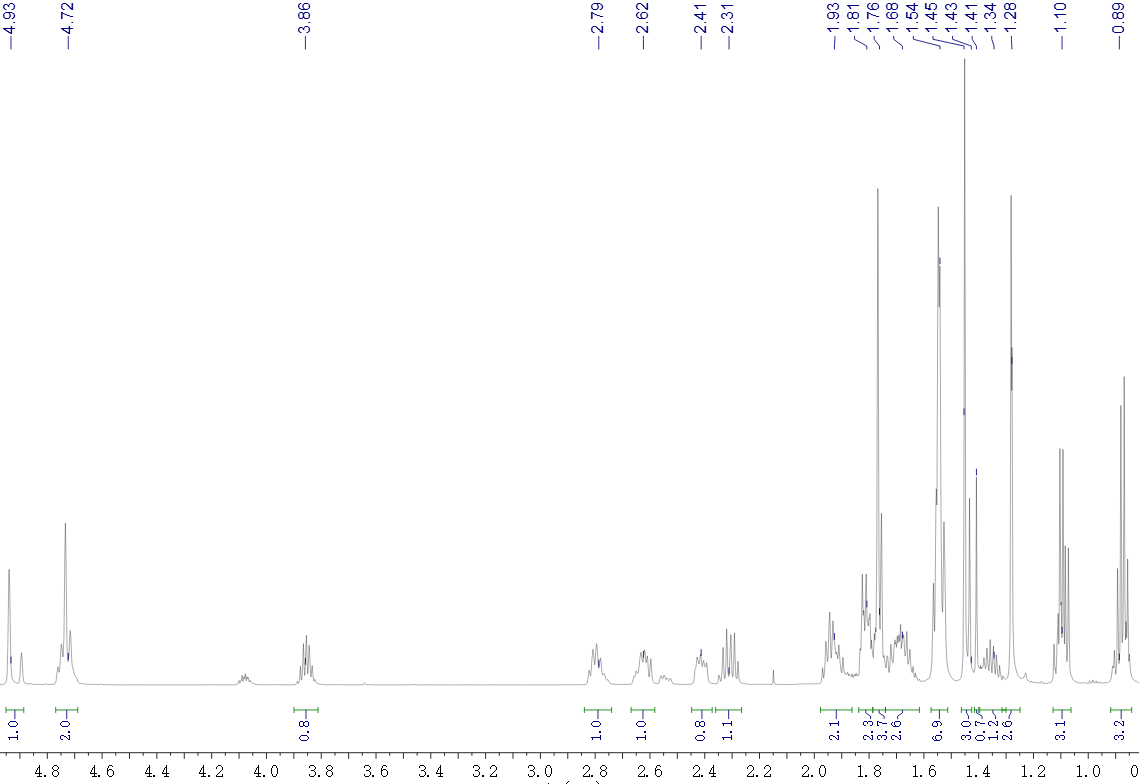


**Fig. S25** ^1^H NMR spectrum of hyperhenol D (**4**) in CDCl_3_


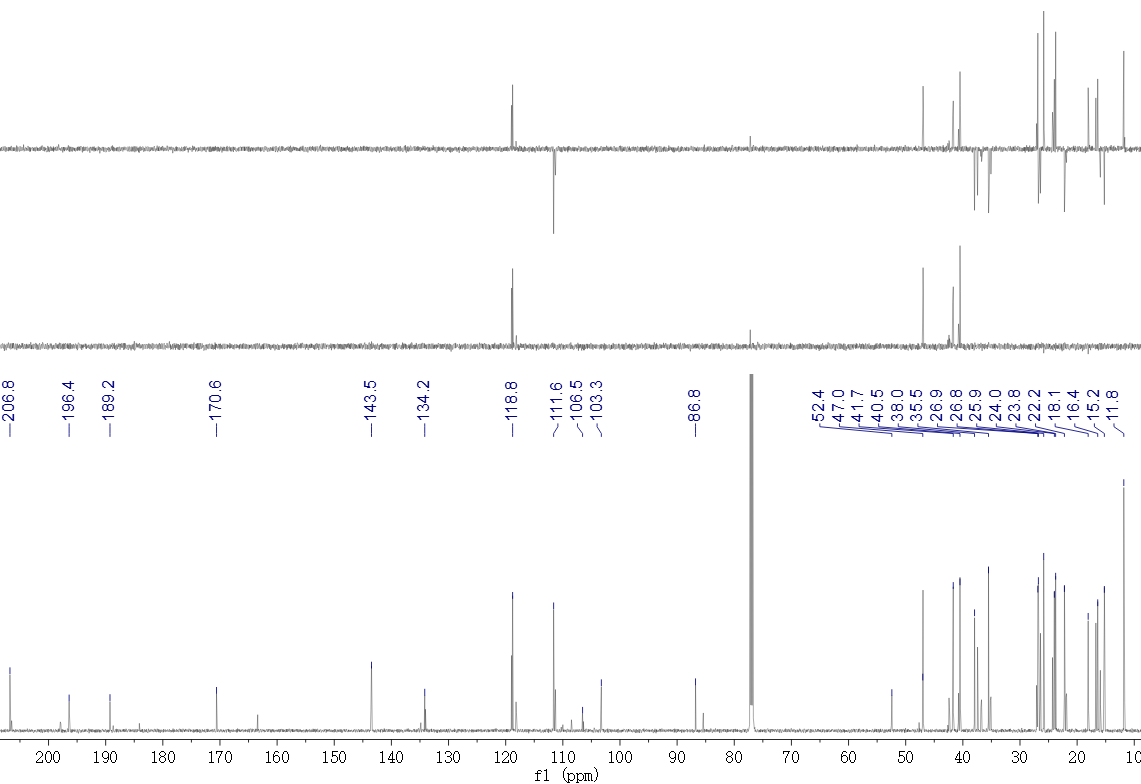


**Fig. S26** ^1^H NMR spectrum of hyperhenol D (**4**) in CDCl_3_


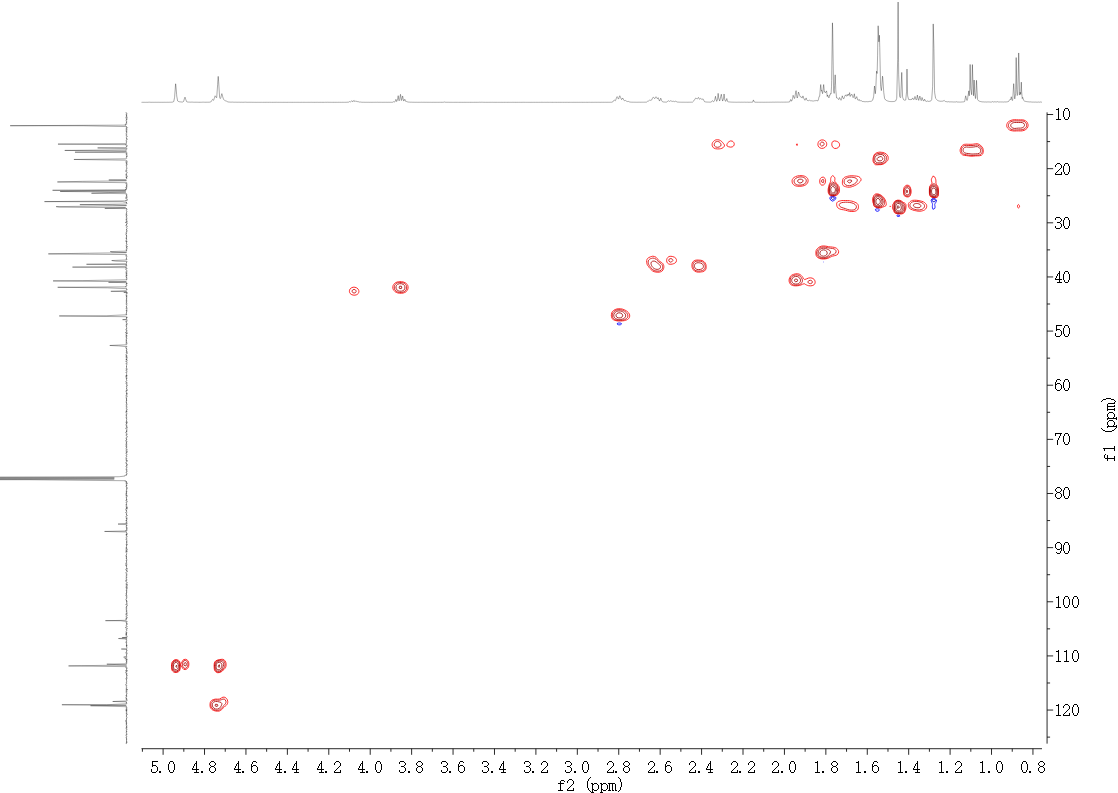


**Fig. S27** HSQC spectrum of hyperhenol D (**4**) in CDCl_3_


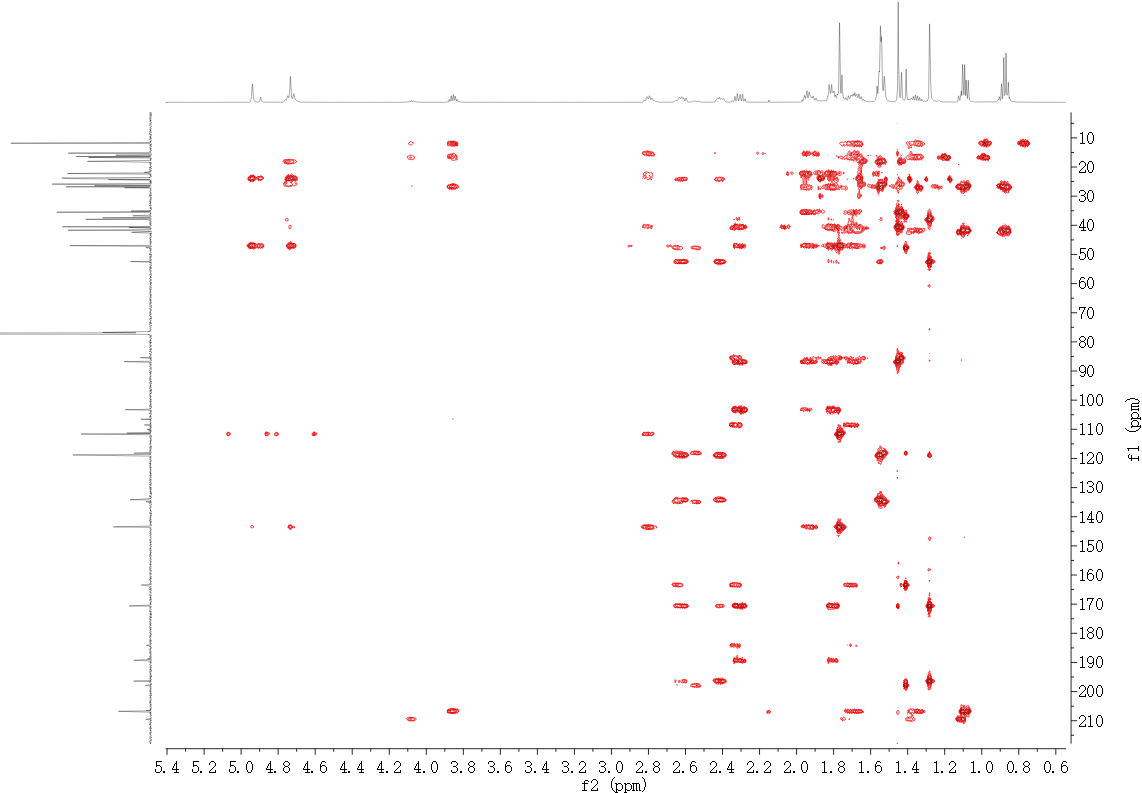


**Fig. S28** HMBC spectrum of hyperhenol D (**4**) in CDCl_3_


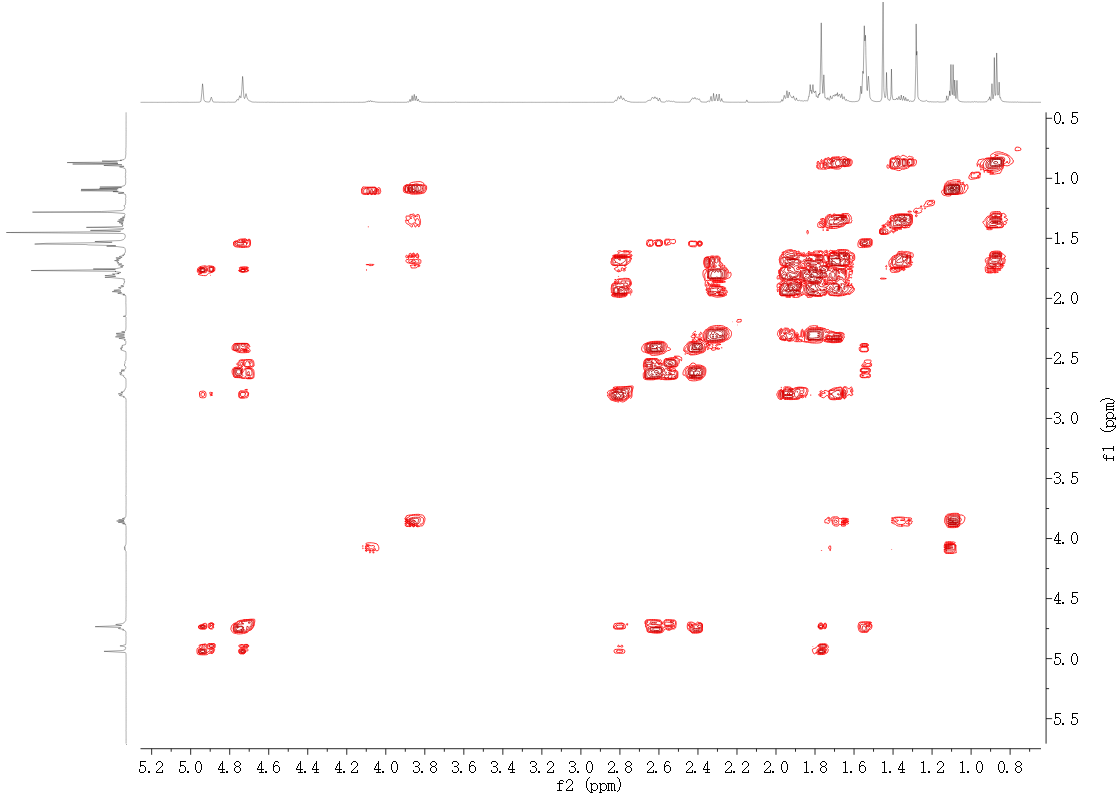


**Fig. S29** ^1^H-^1^H COSY spectrum of hyperhenol D (**4**) in CDCl_3_


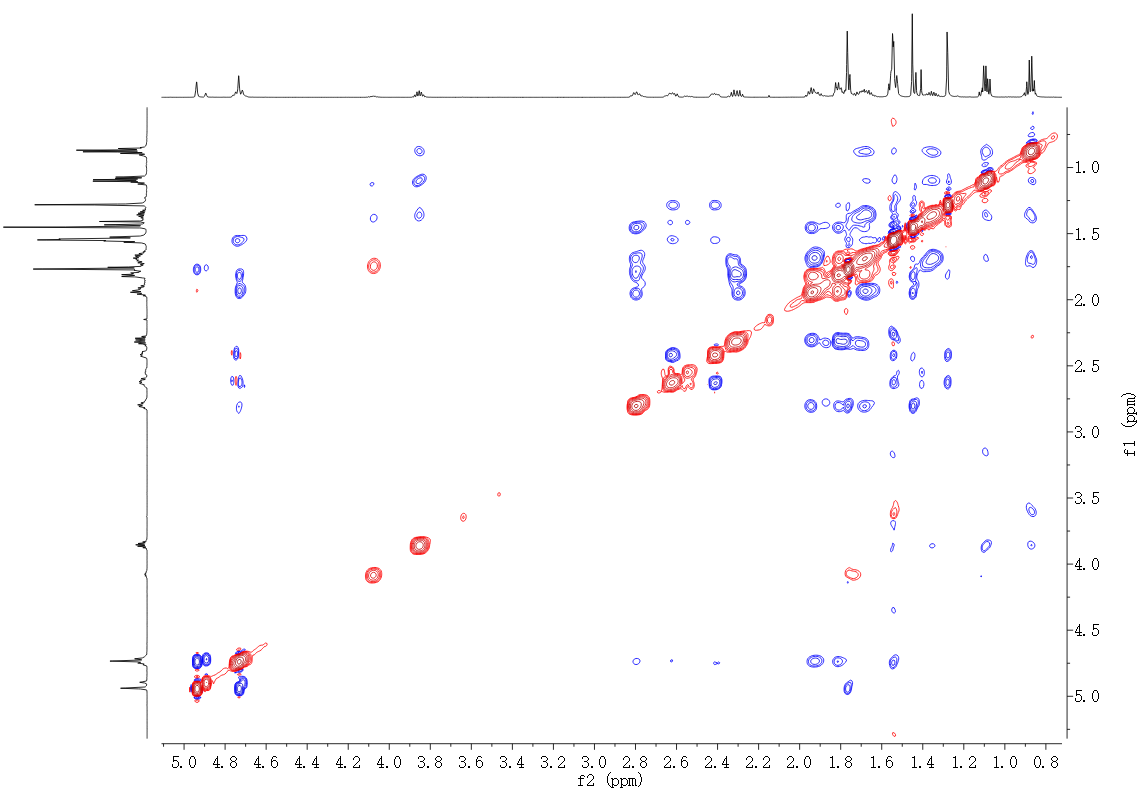


**Fig. S30** ROESY spectrum of hyperhenol D (**4**) in CDCl_3_

#
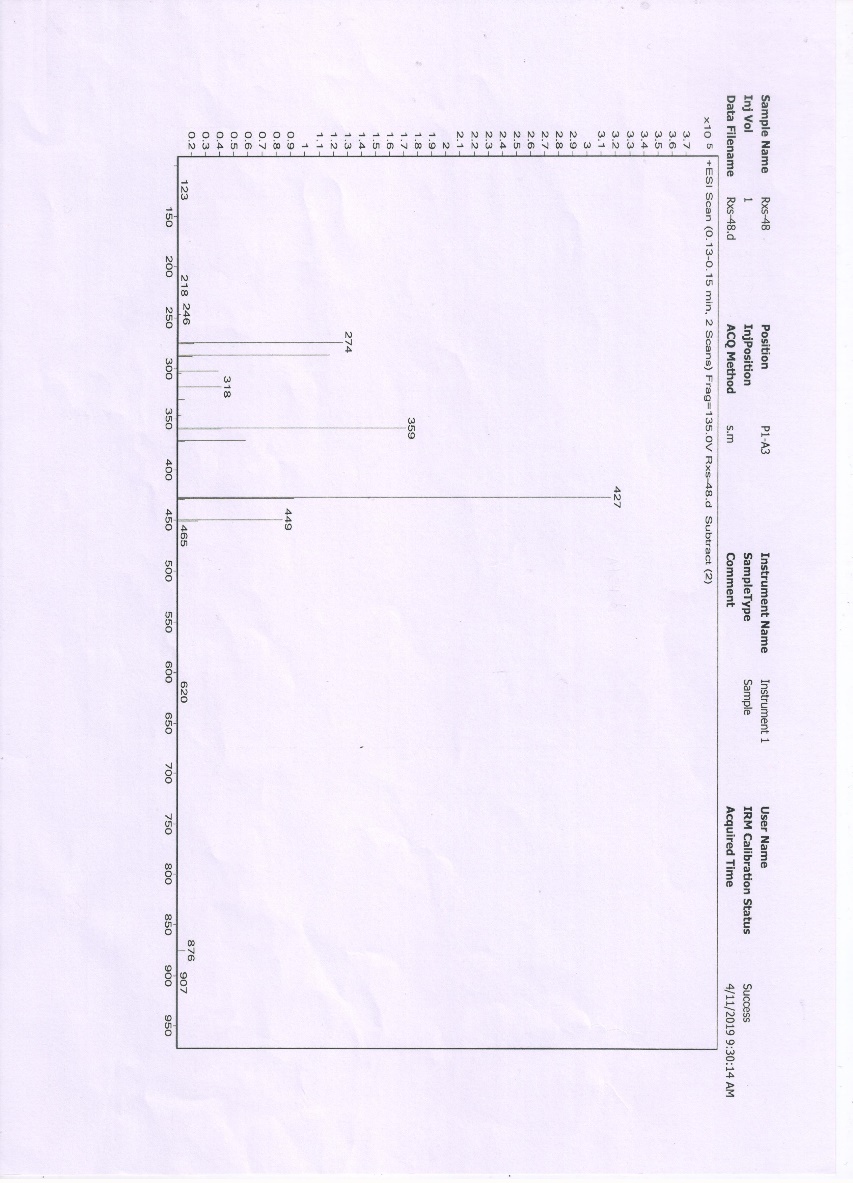


**Fig. S31** ESIMS spectroscopic report of hyperhenol D (**4**)


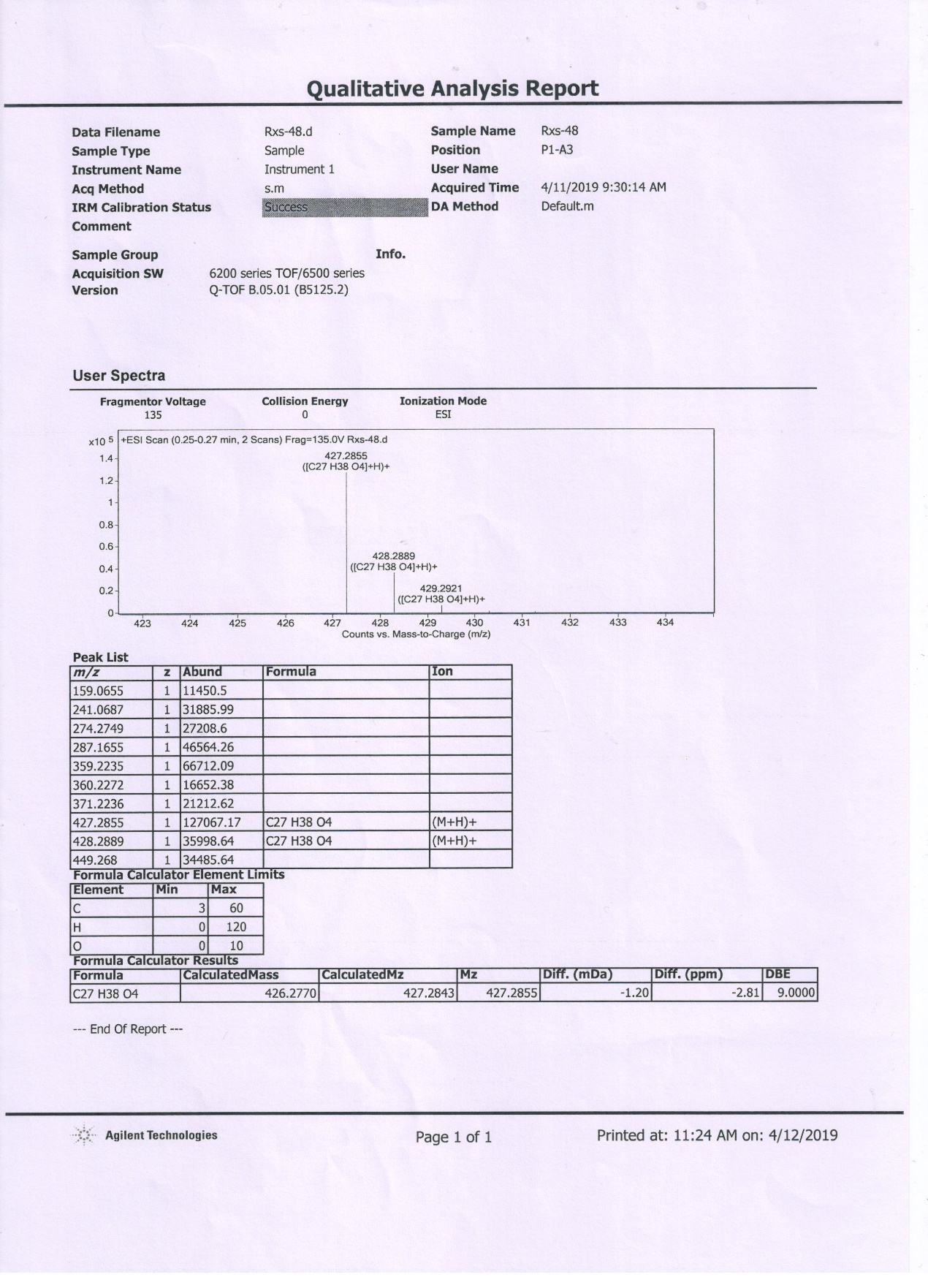


**Fig. S32** HRESIMS spectroscopic report of hyperhenol D (**4**)


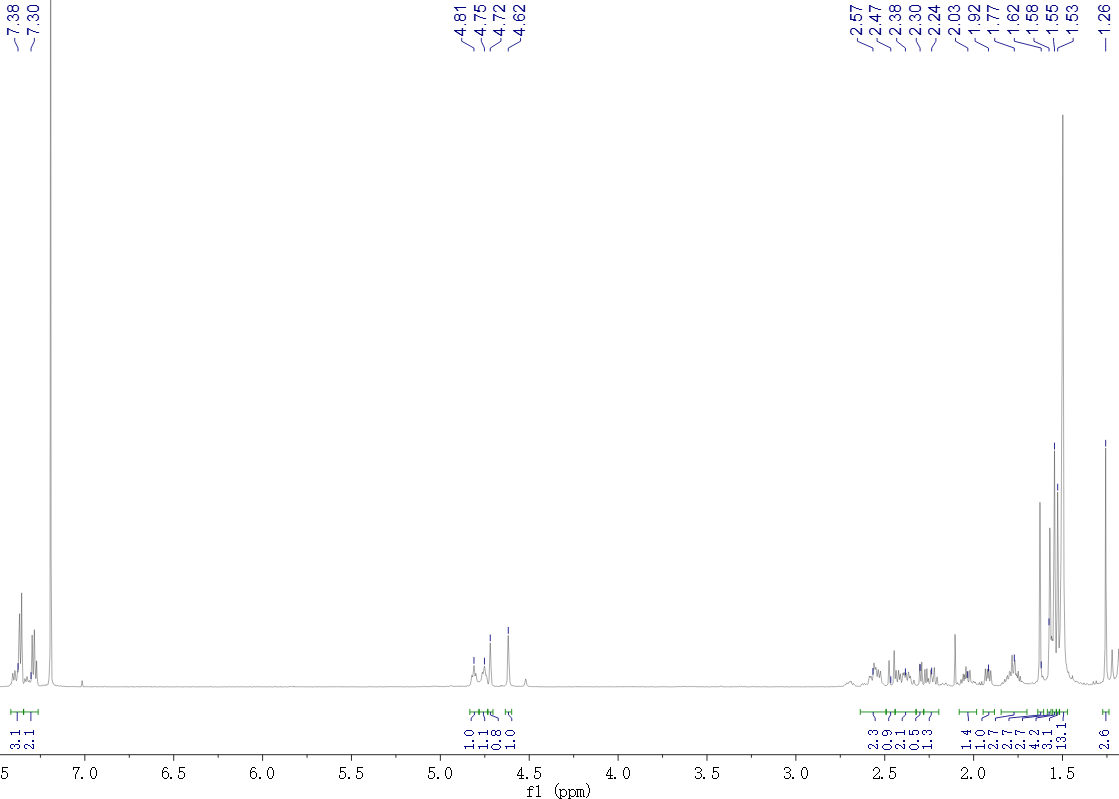


**Fig. S33** ^1^H NMR spectrum of hyperhenolE (**5**) in CDCl_3_


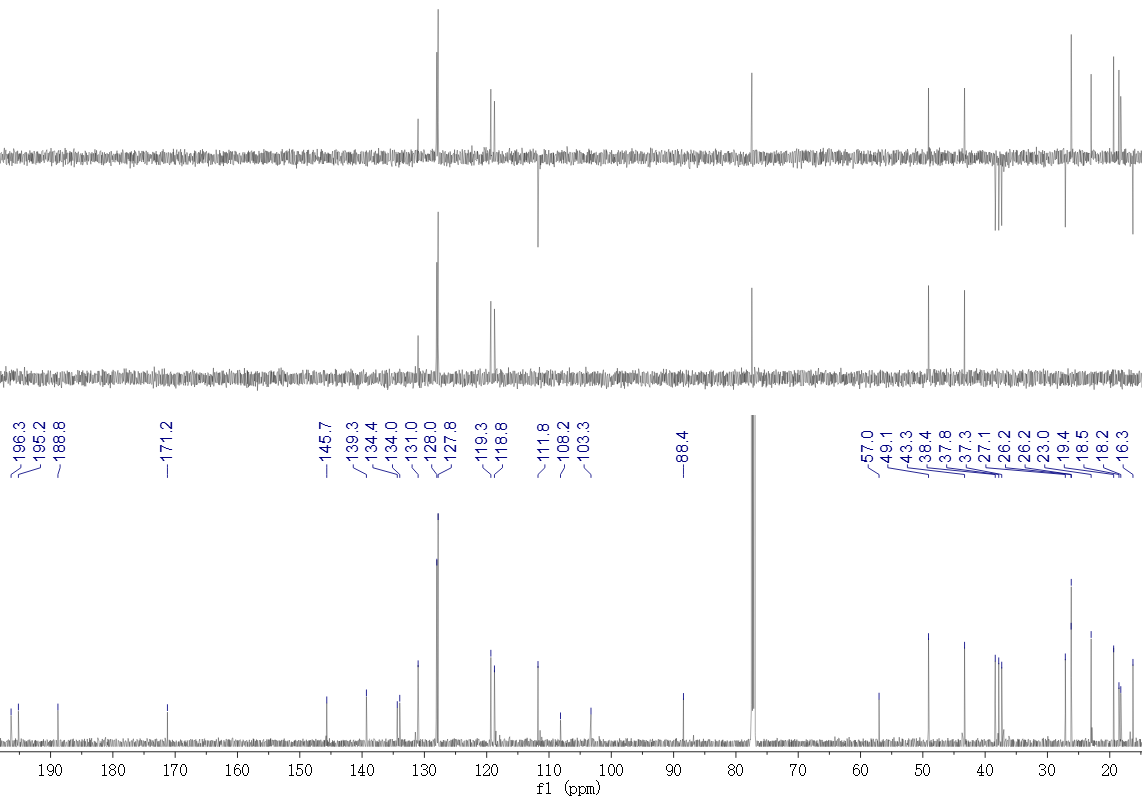


**Fig. S34** ^13^C NMR spectrum of hyperhenol E (**5**) in CDCl_3_
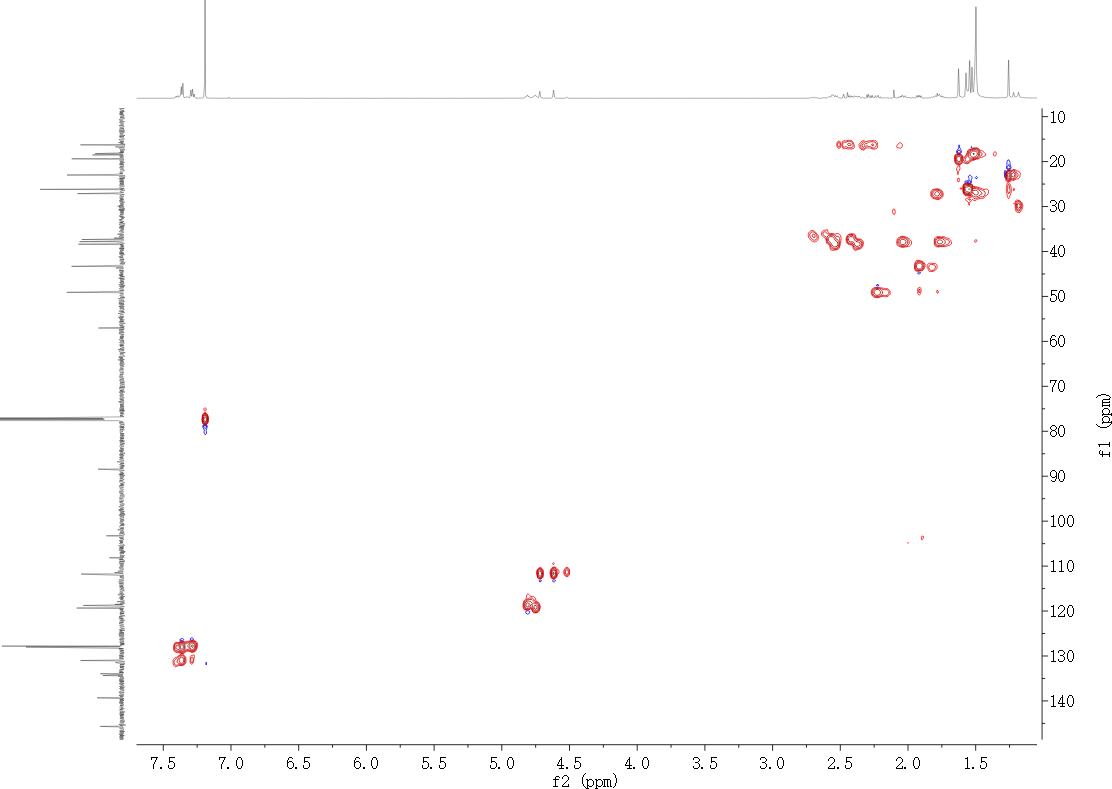


**Fig. S35** HSQC spectrum of hyperhenol E (**5**) in CDCl_3_


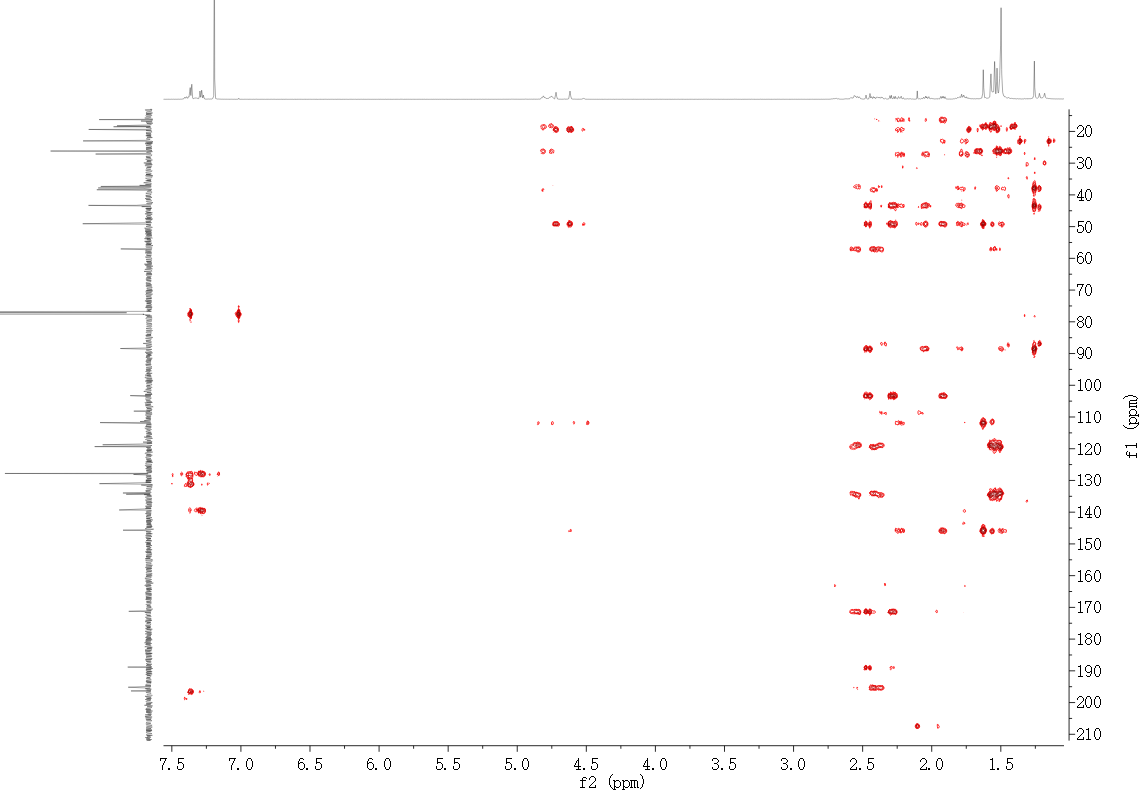


**Fig. S36** HMBC spectrum of hyperhenol E (**5**) in CDCl_3_


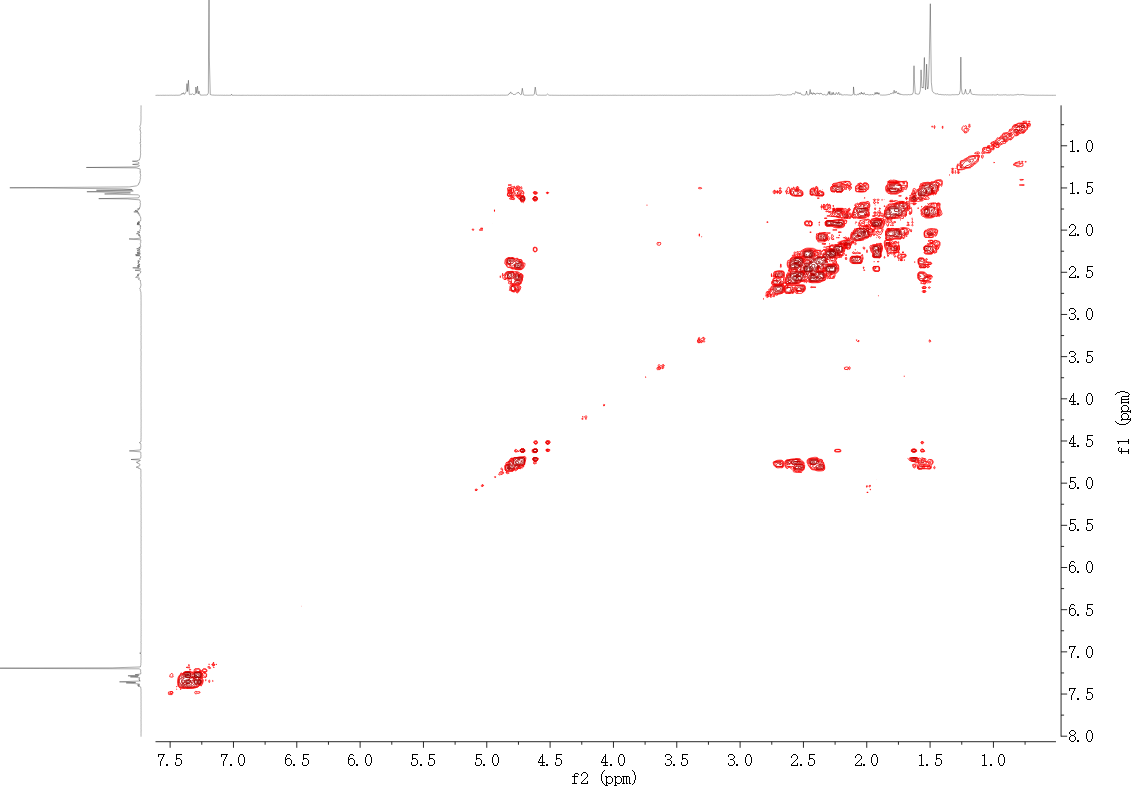


**Fig. S37** ^1^H-^1^H COSY spectrum of hyperhenol E (**5**) in CDCl_3_


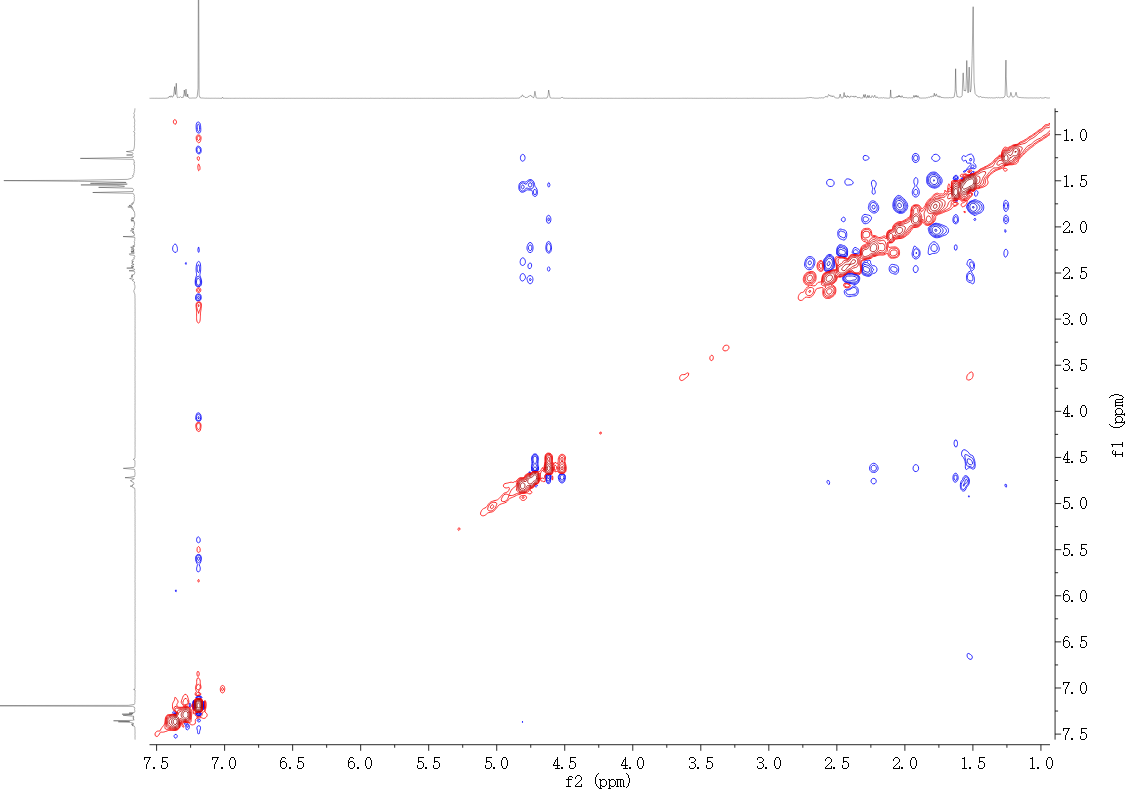


**Fig. S38** ROESY spectrum of hyperhenol E (**5**) in CDCl_3_


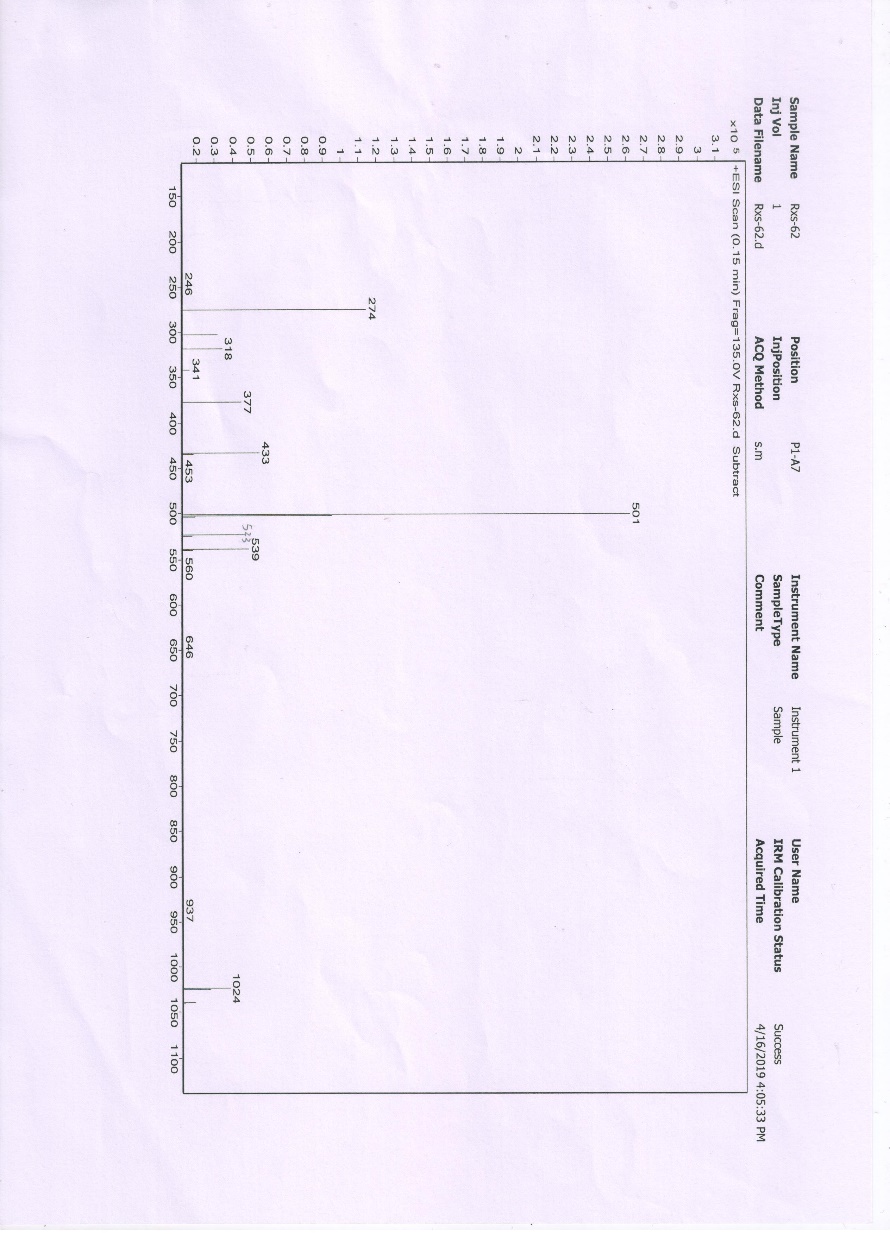


**Fig. S39** ESIMS spectroscopic report of hyperhenol E (**5**)


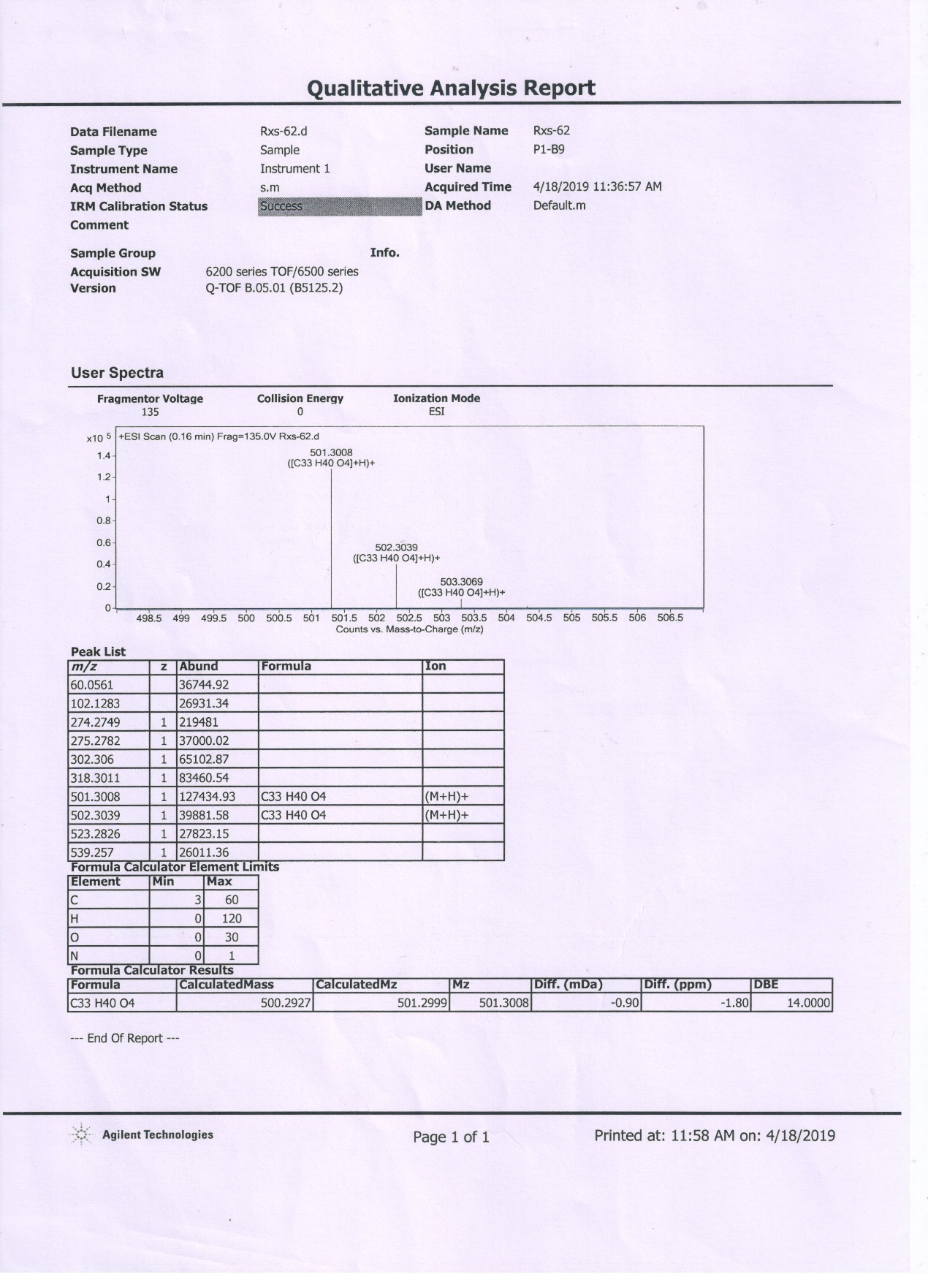


**Fig. S40** HRESIMS spectroscopic report of hyperhenol E (**5**)
